# Transcriptome analysis and identification of chemosensory genes in the larvae of *Plagiodera versicolora*

**DOI:** 10.1186/s12864-022-09079-2

**Published:** 2022-12-22

**Authors:** Zhe-Ran Wu, Jian-Ting Fan, Na Tong, Jin-Meng Guo, Yang Li, Min Lu, Xiao-Long Liu

**Affiliations:** 1 State Key Laboratory of Biocatalysis and Enzyme Engineering, School of Life Sciences, Hubei University, Wuhan, 430062 China; 2 School of Forestry and Biotechnology, Zhejiang A & F University, National Joint Local Engineering Laboratory for High-Efficient Preparation of Biopesticide, Lin’an, 311300 China; 3 Key Laboratory of Integrated Management of Crop Disease and Pests, Ministry of Education/ Department of Entomology, Nanjing Agricultural University, Nanjing, 210095 China

**Keywords:** *Plagiodera versicolora*, transcriptome, odorant binding proteins, chemosensory proteins

## Abstract

**Background:**

In insects, the chemosensory system is crucial in guiding their behaviors for survival. *Plagiodera versicolora* (Coleoptera: Chrysomelidae), is a worldwide leaf-eating forest pest in salicaceous trees. There is little known about the chemosensory genes in *P. versicolora*. Here, we conducted a transcriptome analysis of larvae heads in *P. versicolora*.

**Results:**

In this study, 29 odorant binding proteins (OBPs), 6 chemosensory proteins (CSPs), 14 odorant receptors (ORs), 13 gustatory receptors (GRs), 8 ionotropic receptors (IRs) and 4 sensory neuron membrane proteins (SNMPs) were identified by transcriptome analysis. Compared to the previous antennae and foreleg transcriptome data in adults, 12 OBPs, 2 CSPs, 5 ORs, 4 IRs, and 7 GRs were newly identified in the larvae. Phylogenetic analyses were conducted and found a new candidate CO_2_ receptor (*PverGR18*) and a new sugar receptor (*PverGR23*) in the tree of GRs. Subsequently, the dynamic expression profiles of various genes were analyzed by quantitative real-time PCR. The results showed that *PverOBP31*, *OBP34*, *OBP35*, *OBP38,* and *OBP40* were highly expressed in larvae, *PverOBP33* and *OBP37* were highly expressed in pupae, and *PverCSP13* was highly expressed in eggs, respectively.

**Conclusions:**

We identified a total of 74 putative chemosensory genes based on a transcriptome analysis of larvae heads in *P. versicolora*. This work provides new information for functional studies on the chemoreception mechanism in *P. versicolora.*

**Supplementary Information:**

The online version contains supplementary material available at 10.1186/s12864-022-09079-2.

## Background

Insect chemosensory system mainly includes the olfactory system and gustatory system, which are involved in complex behaviors, such as feeding, mating, ovipositing, and avoiding dangers and enemies [1–4]. The major chemosensory proteins include odorant-binding proteins (OBPs), chemosensory proteins (CSPs), odorant receptors (ORs), gustatory receptors (GRs), ionotropic receptors (IRs), and sensory membrane proteins (SNMPs) [5–11]. In the proposed process of odorant and taste detection, molecules are first bound and transported by OBPs or/and CSPs within the sensillum lymph, then detected by ORs, GRs or/and IRs expressed at the membrane of olfactory sensory neurons. After that, electrical signals are transmitted to the central nervous system through nerve axons inducing an action potential [12–15]. Other protein families, such as SNMPs, are also critical for pheromone detection [16, 17].

The first insect OBP was a pheromone-binding protein that was discovered from an antenna extract of the giant moth *Antheraea polyphemus* [5]. The structure of OBPs is further stabilized by three interlocked disulfide bridges between conserved cysteines [18–20]. The classical OBP fold possesses a core of six α-helices, a member of the C-plus class of OBPs, which has a longer sequence than classical OBPs, and C-minus OBPs have a shorter conserved cysteine [21–24].

The first identified member of the CSP family, called *p10,* was first identified in the American cockroach *Periplaneta americana* [25]*.* Later, a similar protein, OS-D (or A10) proteins, were identified by subtractive hybridization in the antennae of *Drosophila melanogaster* [18]. OBPs and CSPs, belong to a class of small water-soluble proteins and are impregnated in the sensilla lymph [26, 27]. CSPs, instead, seem to form a more homogeneous group of proteins, some with only four instead of six α-helical domains [28]. A large number of OBPs and CSPs have been identified in different insect species, particularly in recent years, due to genome projects and transcriptome sequencing [29–33].

ORs and GRs are ligand-gated cation channels, and encode seven transmembrane domain proteins with an intracellular N terminus and an extracellular C terminus [34, 35]. Insect ORs are composed of two related heptahelical subunits: a divergent conventional ligand-binding OR subunit that confers odor specificity [36, 37] and a highly conserved co-receptor (Orco) subunit [38]. Unlike ORs, GRs can function independently as monomers, such as the sugar receptor [39, 40] or form obligatory heteromers of two receptors as the CO_2_ receptor [41]. Insect IRs act as ligand-based ion channels that are related to ionotropic glutamate receptors [42]. Similar to ORs, the antennal IRs form heteromeric complexes with one or more co-receptors (IR8a, IR25a or IR76b) to perform their functions [43, 44].

The willow leaf beetle *Plagiodera versicolora* (Coleoptera: Chrysomelidae), is a leaf-eating forest pest, which mainly damages salicaceous trees, such as willows (*Salix*) and poplars (*Populus*) [45, 46]. In our previous study, we obtained 111 chemosensory genes from antennae [47] and forelegs transcriptomes [48], including 41 ORs, 28 OBPs, 11 CSPs, 17 GRs, 10 IRs, and 4 SNMPs. Antennae and maxillae are the paired principal chemosensory organs located on the head of the larvae [49]. Considering that the head is the tissue where most olfactory and gustatory sensilla are located, we identified more chemosensory genes from the larvae head transcriptomes of *P. versicolora*. Phylogenetic analysis was conducted among these chemosensory genes and other insect species. The quantitative real-time PCR was utilized in our study to screen the expression patterns. This work could provide a basis to facilitate functional clarification of these chemosensory genes at the molecular level.

## Methods

### Insect rearing and tissue collection

The larvae and adults of *P. versicolora* were collected from Sha Lake Park in Wuhan, Hubei Province, and maintained at the laboratory following conditions at 28 ± 1 °C, 70% ± 5% relative humidity, and a 12 h: 12 h light: dark (L: D) photoperiod. Larvae and adults were fed with a fresh leaf of willows. A total of 150 third instar larvae heads were collected and the tissues were immediately frozen in liquid nitrogen and stored at −80 °C for RNA extraction.

### RNA extraction, cDNA Library Construction and Sequencing

The total RNA of the third instar larvae heads was extracted using TRIzol reagent (Invitrogen, Carlsbad, CA, USA) following the manufacturer’s instruction. RNA integrity was verified by gel electrophoresis. RNA concentration was measured on a Nanodrop ND-2000 spectrophotometer (NanoDrop products, Wilmington, DE, USA). Three biological replicates were used in the study. A total RNA of about 2 μg of each sample was used to construct the cDNA libraries. The libraries were sequenced using the Illumina Novaseq 6000 and performed at Shanghai Majorbio Bio-pharm Technology Co., Ltd. following the detailed protocol.

### Transcriptome assembly and annotation

Datasets of clean reads were obtained by removing adaptor reads, ploy-N and low-quality reads, from the raw data and were further assembled into transcripts using Trinity v2.5.1 with default parameters [50]. Functional annotation of the longest transcript sequence (unigenes) was performed against six databases, non-redundant database (Nr), Pfam, Clusters of Orthologous Groups (COG), Swiss-Prot, Kyoto Encyclopedia of Genes and Genomes (KEGG), and Gene Ontology (GO), respectively.

### Chemosensory Gene Identification and phylogenetic tree construction

To identify putative chemosensory genes (ORs, GRs, IRs, OBPs, CSPs, and SNMPs), we search the results of non-redundant protein (Nr) annotation from our transcriptome dataset. Annotations of all retrieved sequences were confirmed by blastx on the National Center for Biotechnology Information (NCBI). The Open reading frames (ORFs) of all putative chemosensory genes were predicted by ORF finder (https://www.ncbi.nlm.nih.gov/orffinder/). The transmembrane domains of ORs, IRs and GRs were predicted by TMHMM2.0 (https://services.healthtech.dtu.dk/service.php? TMHMM-2.0). Putative N-terminal signal peptides of OBPs and CSPs were predicted using SignalP 5.0 (https://services.healthtech.dtu.dk/service.php? SignalP-5.0).

Phylogenetic analyses were performed based on amino acid sequences from candidate chemosensory genes in *P. versicolora* and other insects. For the OBPs and CSPs tree sets, we selected the protein sequences including four Coleoptera from different families (Table S1), *Colaphellus bowringi* [51], *Monochamus alternatus* [52], *Dastarcus helophoroides* [52] and *Ophraella communa* [53]. For the GRs tree, the protein sequences from two Coleoptera (Chrysomelidae) species (*C. bowringi* and *O. communa*), the sequences that have been functionally identified, including three Lepidoptera (*Plutella xylostella* [54], *Helicoverpa armigera* [51, 55, 56] and *Bombyx mori* [35, 39, 57]) and four Diptera (*D. melanogaster* [40, 58–60], *Anopheles gambiae* [61], *Aedes aegypti* [41] and *Anopheles coluzzii* [62]) were used. ClustalW (gap opening penalty: 10, gap extension penalty: 0.2) was employed for amino acid sequence alignment and the neighbor-joining method was constructed using MEGA6, the gap/missing data treatment was set as partial deletion with a 95% site coverage cutoff [63, 64]. Branch support was assessed with 1000 bootstrap replicates. Phylogenetic trees were visualized and edited by FigTree 1.4.3(http://tree.bio.ed.ac.uk/software/figtree/).

### Expression analysis by quantitative real-time PCR

Quantitative real-time PCR (qRT-PCR) was performed to verify the expression patterns of candidate chemosensory genes by using CFX Connect Real-Time System (Bio-Rad, CA, USA). Different stages, including eggs, first instar larvae, second instar larvae heads, third instar larvae heads and pupae, were collected. Total RNA was isolated using the methods described above and reverse transcribed into cDNA using HiScript® III RT SuperMix for qPCR (+gDNA wiper) (Vazyme, Nanjing, China). The qRT-PCR was conducted in a 20 μL reaction system, containing 10 μL ChamQ Universal SYBR qPCR Master Mix (2×), 0.4 μL each of the forward and reverse primers (10 μM), 1 μL cDNA template, and 8.2 μL of nuclease-free water. The reaction conditions were as follows: 95 °C for 30 s, 40 cycles of 95 °C for 5 s and 60 °C for 30 s. Gene expression profiles were analyzed using the 2 ^−ΔΔCT^ method [65]. The means and variances of the treatments were analyzed in a one-way ANOVA using SPSS 26.0 software. Each reaction for each tissue was performed in three biological replicates. The *RPS18* gene (accession number: OM885975) was used as the reference gene for normalizing the expression of various samples [66]. And *RPS18* gene has been proven stable in different development stages, female and male adults, different tissues, different temperatures, and pathogen treatment [67]. The gene-specific primers were designed using Beacon Designer 8.0 (amplicon length: 100 ± 20 bp; Tm: 60 ± 2 °C; GC%: 45–55%) (PRIMER Biosoft International, CA, USA), and were listed in Supplementary Table S2.

## Results

### Transcriptome sequencing, assembly and analysis

To identify the chemosensory genes of *P. versicolora*, the transcriptome sequencing of third instar larvae heads was sequenced with three independent biological replicates. Approximately 75.14 million, 68.24 million and 77.56 million raw reads, and a total of 74.50 million, 67.73 million and 76.97 million clean reads were generated. Clean reads were assembled into 28,268 unigenes with an average length of 1128 bp, and an N50 of 2500 bp. Length distribution analysis showed that 45.35% of all unigenes were longer than 500 bp in size (Fig. S1).

After function annotations, a total of 10,030 unigenes (35.74%) and 13,310 unigenes (47.43%) were successfully annotated through GO and Nr datasets, respectively. In the GO analysis, the unigenes were assigned to three main functions: biological process (13,448), cellular component (16,640), and molecular function (12,036). Within three classes, the subcategories “binding” (5321), “cell part” (4857) and “cellular process” (4475) contained the majority of the unigenes, respectively (Fig. S2).

### Identification of candidate OBPs

**Fig. 1:**
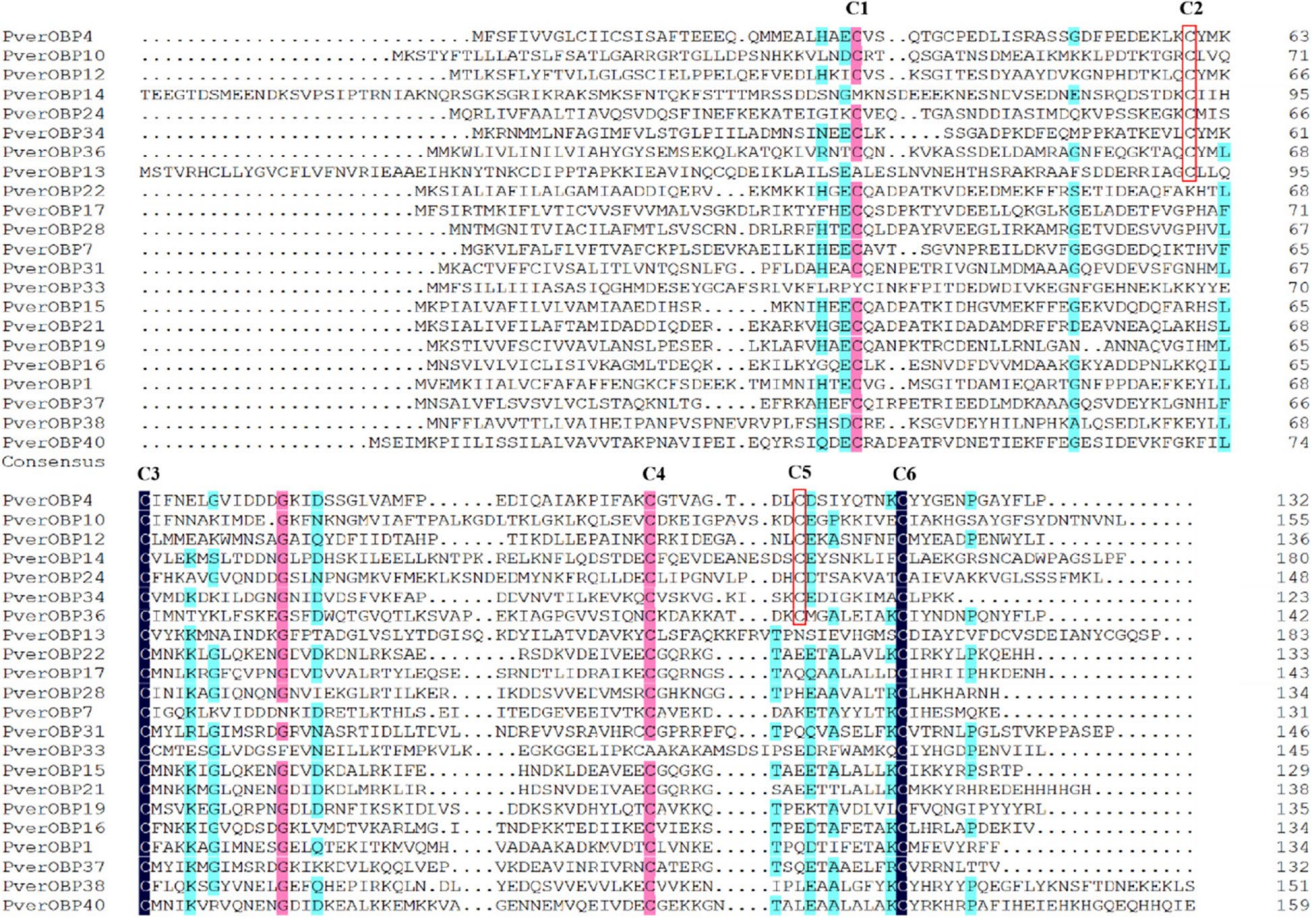
Multiple amino acid alignments of the predicted Classic OBPs and Minus-C OBPs. Conserved cysteine residues are marked with a red box (C2 and C5), black (C3, C4 and C6) and pink (C1) background. The number of 61 and 107 amino acids were deleted at the begin of OBP14 and end of OBP38, respectively

**Fig. 2:**
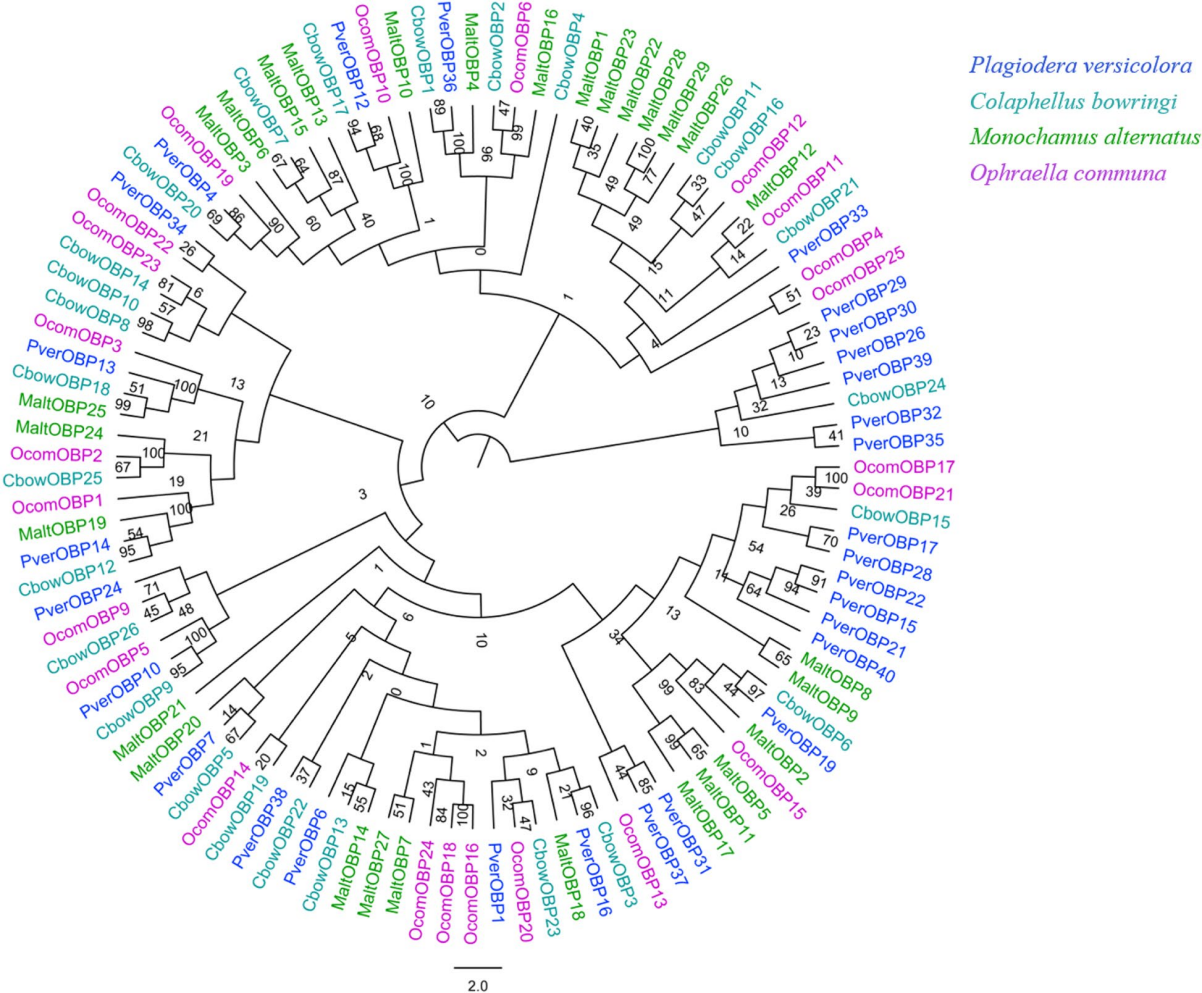
Phylogenetic analysis of the OBPs (odorant-binding proteins) from four insect species: *P. versicolora* (Pver), *C. bowringi* (Cbow), *M. alternatus* (Malt), *O. communa* (Ocom). The *P. versicolora* genes are shown in blue. The values at the branch nodes represent bootstrap values based on 1000 replicates

In this study, a total of 29 predicted PverOBP transcripts were identified from third instar larvae heads transcriptomes, 12 PverOBPs were new compared to antennae and forelegs. The complete ORF was detected in 22 PverOBPs, with lengths ranging from 131 to 241 amino acids. Among them, 22 transcripts with the complete sequence have a signal peptide sequence (Table S3). Sequence analysis showed that 6 PverOBPs belonged to the Classical OBP subclass with the typical six conserved cysteine residues. Fifteen PverOBPs belonged to the minus-C OBP subgroup with 4 conserved cysteine residues (Fig. 1). A phylogenetic analysis showed that *PverOBP4* and *OBP12* were on the same branch as *MaltOBP3* and *OBP10*, which functions were identified in *M. alternatus*, respectively (Fig. 2).

### Identification of candidate CSPs

**Fig. 3:**
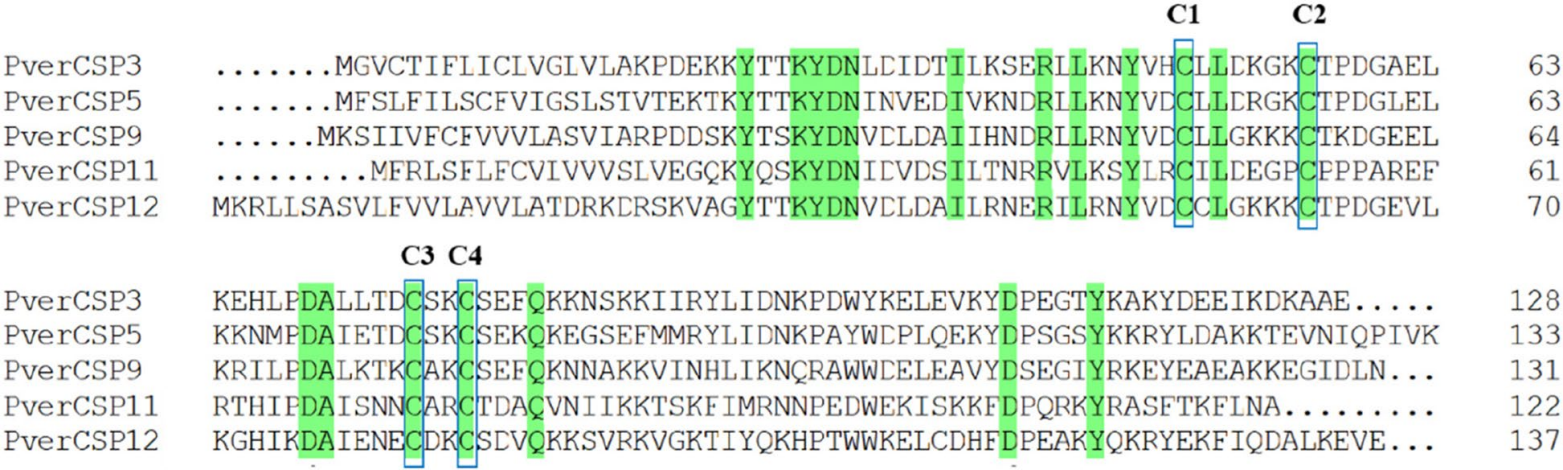
Multiple amino alignments of the predicted CSPs. Conserved cysteine (C1-C4) residues are marked with a blue box

**Fig. 4:**
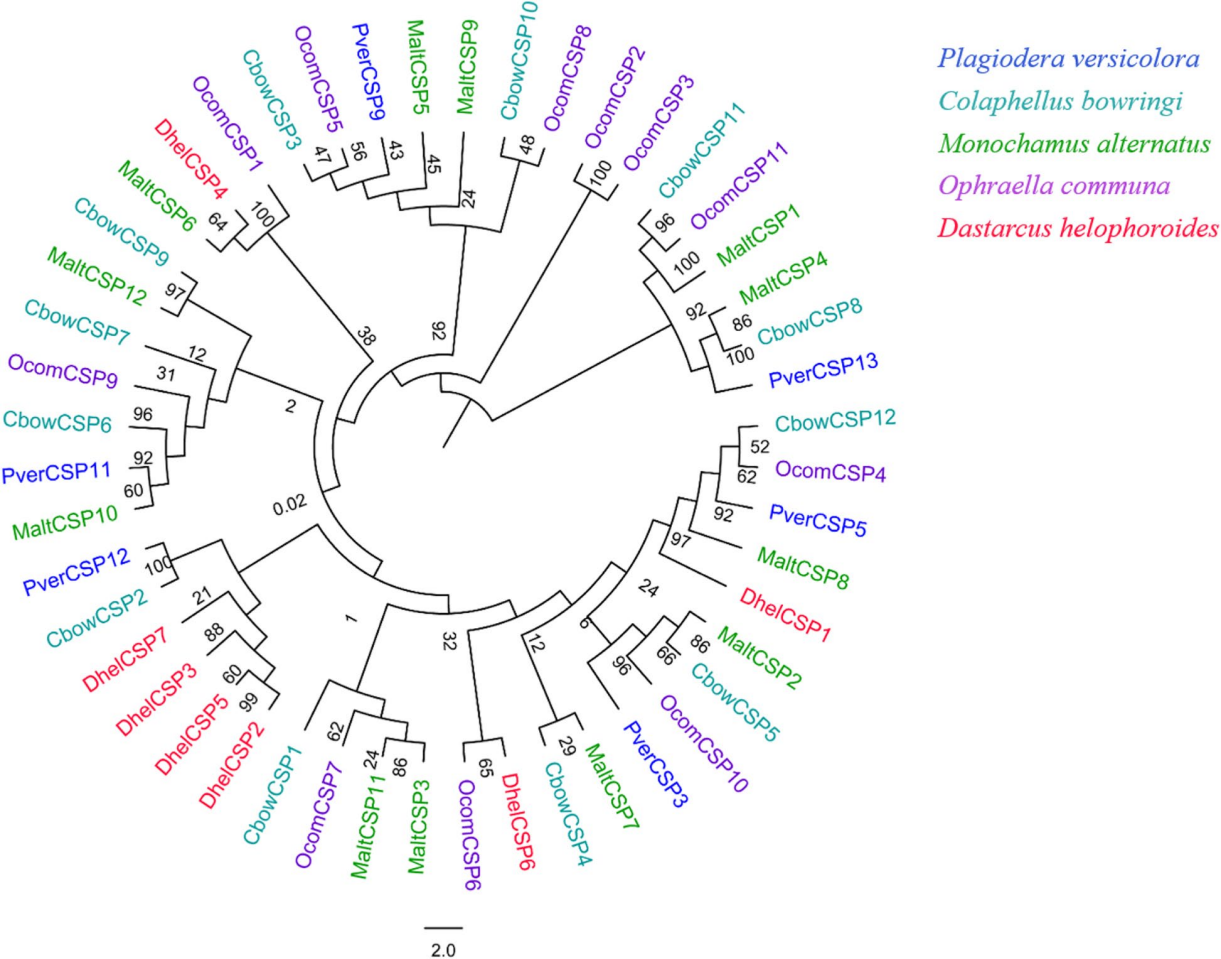
Phylogenetic analysis of the CSPs (chemosensory proteins) from four insect species: *P. versicolora* (Pver), *C. bowringi* (Cbow), *M. alternatus* (Malt), *O. communa* (Ocom) and *D. helophoroides* (Dhel). The *P. versicolora* genes are shown in blue. The values at the branch nodes represent bootstrap values based on 1000 replicates

Six transcripts encoding CSPs were identified from the *P. versicolora* transcriptomes and *PverCSP12* and *CSP13* were newly compared to antennae and forelegs transcriptomes. All but one transcript (PverCSP13) included full-length ORFs, with lengths from 122 to 137 amino acids and a predicted signal peptide. Five CSP proteins have four highly conserved cysteine residues, which are characteristic of typical insect CSPs (Fig. 3). Phylogenetic analyses of CSPs from *P. versicolora* and other four Coleoptera species showed that *PverCSP9* was clustered with *MaltCSP5* in *M. alternatus* that functional characteristics have been performed (Fig. 4).

### Identification of candidate GRs

**Fig. 5:**
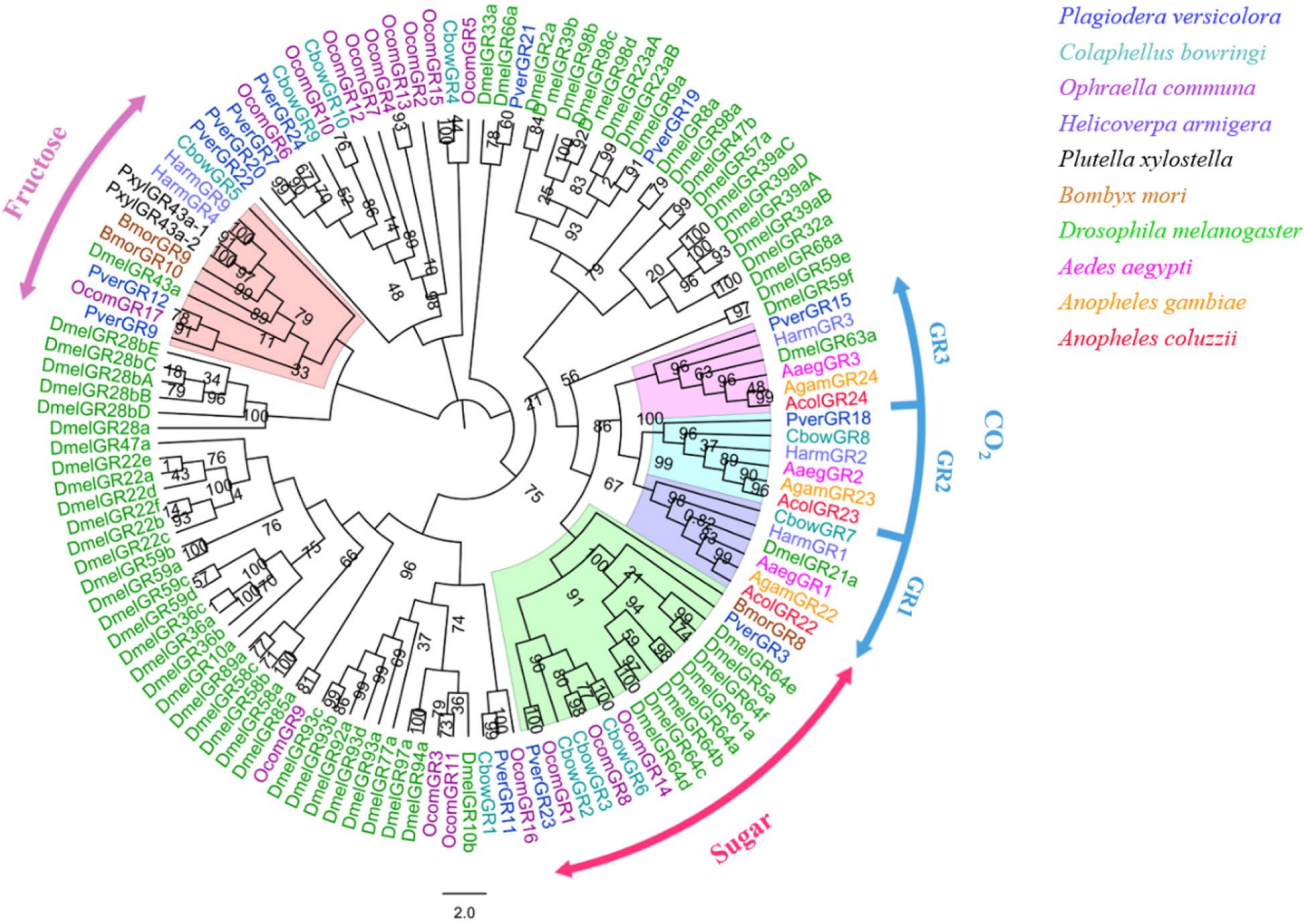
Phylogenetic analysis of the GRs (gustatory receptors) from ten insect species: *P. versicolora* (Pver), *C. bowringi* (Cbow), *O. communa* (Ocom), *A. gambiae* (Agam), *A. aegypti* (Aage), *A. coluzzii* (Acol), *D. melanogaster* (Dmel), *H. armigera* (Harm), *P. xylostella* (Pxyl) and *B. mori* (Bmor). The *P. versicolora* genes are shown in blue. The values at the branch nodes represent bootstrap values based on 1000 replicates

Thirteen PverGR transcripts were obtained from *P. versicolora* third instar larvae heads and 7 PverGRs were newly identified. Among 13 PverGRs, only *PverGR15* has a full-length ORF, with 7 transmembrane domains (TMDs), which are characteristic of typical insect GRs. Based on the phylogenetic tree analysis that *PverGR15* and *GR18* (newly identified) were clustered in carbon dioxide (CO_2_) receptors, *PverGR3* and *GR23* (newly identified) were clustered in sugar receptors (except fructose). In addition, *PverGR9* and *GR12* were clustered with the reported fructose receptors (Fig. 5).

### Identification of candidate ORs and IRs

From the assembled transcripts, 14 of them were annotated encoding ORs, and 5 PverORs were newly identified from the transcriptomes of third instar larvae heads. The Orco gene was also obtained in the transcriptomes (Table S4). None of these OR unigenes have full-length ORFs, encoding lengths from 45 to 289 amino acids. We identified 8 transcripts encoding candidate IRs in the *P. versicolora* transcriptome and 4 PverIRs were newly identified, compared to antennae and forelegs transcriptomes. These IRs were confirmed belong to IR family by the blast and phylogenetic analysis (Fig. S3). Of these, 3 PverIRs contained a putative full-length ORF, with three to four TMDs.

### Specific expression profiles of candidate genes

**Fig. 6:**
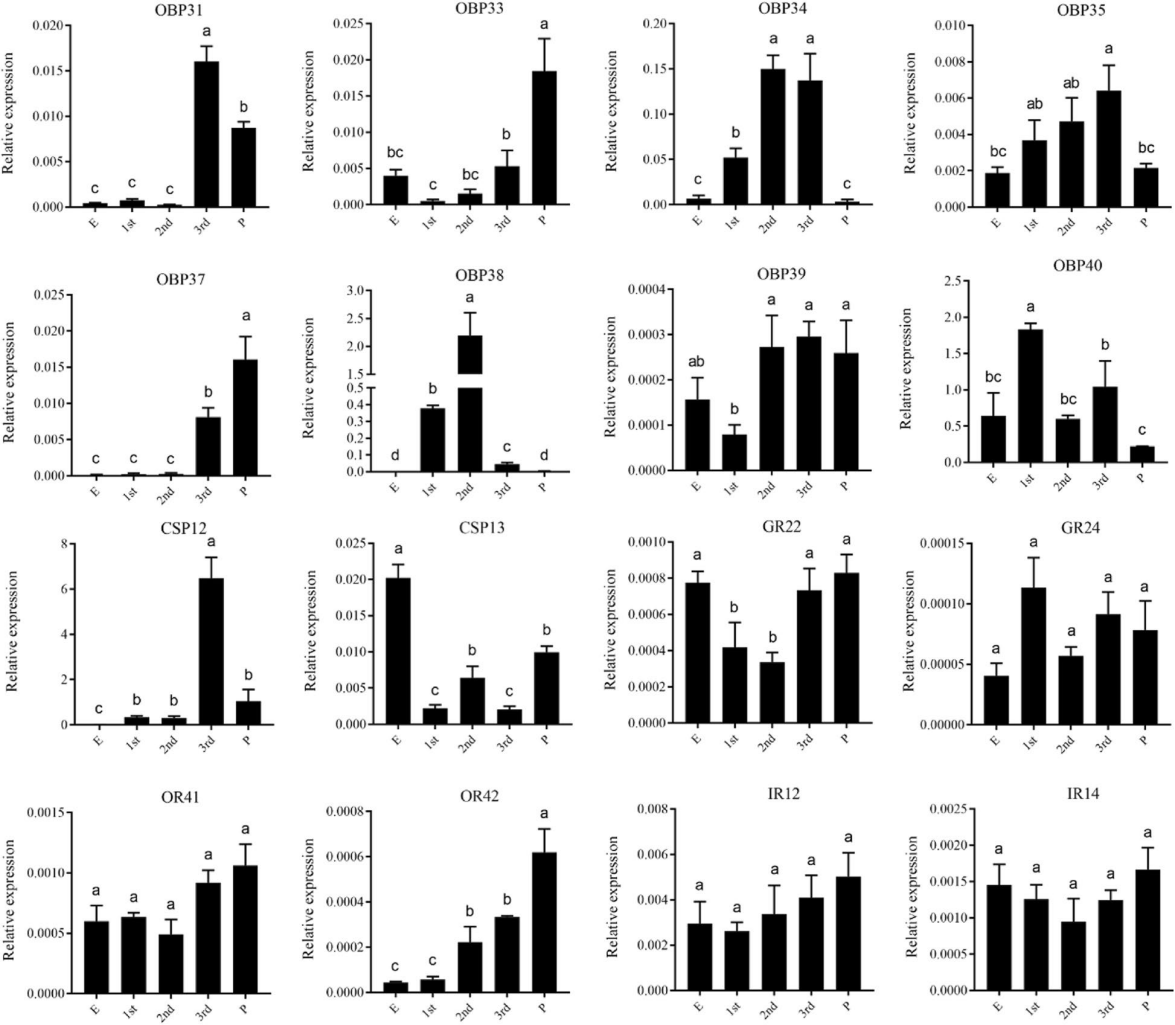
Expression levels of chemosensory genes in different stages performed by qRT-PCR. E, eggs; 1st, first instar larvae; 2nd, second instar larvae heads; 3rd, third instar larvae heads; P, pupae. Error bars indicate standard error of three biological replicates

The qRT-PCR was used to measure the specific expression levels of candidate chemosensory genes during the eggs, larvae and pupae stage (Fig. 6). The PCR efficiencies of primers ranged from 95–105% with high correlation coefficient (R^2^) values (0.991–0.999). *PverOBP31*, *OBP34*, *OBP35*, *OBP38* and *OBP40* were significantly higher expressed in 1st to 3rd instar larvae, while *PverOBP33* and *OBP37* were significantly higher expressed in pupae. *PverCSP12* and *CSP13* have significantly higher expression in 3rd instar larvae and eggs, respectively. *PverGR22* has significantly higher expression in eggs, 3rd instar larvae and pupae, while *PverGR24* has no significant difference in the tested tissues. *PverOR42* was significantly higher expressed in pupae, moderately in 2nd and 3rd instar larvae, while the expression level of *PverOR41* was no significant difference among eggs, larvae and pupae. Both *PverIR12* and *IR14* were no significant difference among eggs, larvae and pupae.

## Discussion

Compared to Dipterans and Lepidopterans, the molecular basis of chemoreception in Coleopterans is relatively poorly understood. In the current study, we sequenced and analyzed the transcriptome of the third instar larvae heads from *P. versicolora*. In total, 74 chemosensory genes were identified, including 29 OBPs, 6 CSPs, 14 ORs, 13 GRs, 8 IRs and 4 SNMPs. Compared to our previous antennae and forelegs transcriptome data in adults, 12 OBPs, 2 CSPs, 5 ORs, 4 IRs and 7 GRs were newly identified (Fig. S4). Moreover, the expression profile of 16 chemosensory genes in different tissues was validated by qRT-PCR, which would facilitate the exploration of the function of these genes. Systematic research on chemosensory genes in larvae may provide valuable information on understanding the molecular mechanisms of insect chemosensory systems.

The phylogenetic tree of OBPs and CSPs of *P. versicolora* was constructed using various Coleoptera species of *C. bowringi*, *M. alternatus* and *O. communa*. *PverOBP4*, *PverOBP12* and *PverCSP9* were clustered with *MaltOBP3* (bound the beetle- or host-plant-related compounds) [68], *MaltOBP10* (bound the volatiles from pine bark) [69] and *MaltCSP5* (bound the odor molecules of pine volatiles) [70], respectively, suggesting that PverOBPs and PverCSPs may have functions (detect the host-plant-related volatiles) similar to these of OBPs and CSPs. Notably, *PverOBP26* (identified in forelegs transcriptome) and five PverOBPs (*PverOBP29*, *30*, *32*, *35* and *39*) formed a cluster with *CbowOBP24*, indicating that these genes of the clade may have suffered gene duplication.

The phylogenetic tree of GRs of *P. versicolora* was constructed using different species, including Coleoptera, Lepidoptera and Diptera. Insect GRs usually have three CO_2_ receptors (*GR1*, *GR2* and *GR3*) family [61, 62, 71], and a new CO_2_ receptor (*PverGR18*), belonging to *GR2* family, was identified from our larvae transcriptome. Considering three CO_2_ receptors have been identified, the function of these putative CO_2_ receptors genes could elucidate in the future. *BmGr10* plays an important role in the myo-inositol recognition required for *B. mori* larval feeding behavior [57]. *PverGR9* and *GR12* were homologues to *BmGr10*, indicating three PverGRs were expressed in larval heads may guide several chemosensory behaviors. *OR41* and *OR52* were obtained from larval antennae and maxillae transcriptome in *H. armigera*, and responded to a four-component blend that strongly attracted larvae [72]. It meant that some ORs were identified in larval heads play role in chemosensory-guided behaviors. However, no PverORs were homologues to *HarmOR41/OR52* (Fig. S5), as the function of 5 PverORs need to identify in the future.

OBPs are commonly postulated to play an important role in the perception of environmental volatiles, in both adult and larval stages [73, 74]. *PverOBP31* and *OBP34* have a highly expression level in the third instars larval heads, and *PverOBP35* showed increased expression levels from the first to third instars. Usually, higher instar larvae of agriculture and forestry pests are crucial for crop and tree feeding [45, 75]. The studies have indicated that *PverOBP31*, *PverOBP34* and *PverOBP35* may participate in larval food searching.

Some pieces of evidence inciting that a relatively simple system, OBPs, abundantly expressed in the larval antennae, also involves the detection of pheromones in larvae [76, 77]. *In vitro* expression and ligand-binding of *SexiOBP13* (highly expressed in the larval heads, and showed increased expression levels from the first- to third-instars), the result showed that it displayed a high binding affinity to Z9, E12–14:Ac, the major sex pheromone component of *S. exigua*, while low affinities to the tested host plant volatiles [78]. In general, PBPs have significantly higher expression in the antennae of adults. This report on *S. exigua* provides a new idea for identifying the function of PBPs in *P. versicolora* that may have a significantly higher expression level (*PverOBP31*, *34*, *35*, *38* and *40*) in larvae. In addition, these five PverOBPs may detection of any other compounds, such as host or non-host volatiles.

Two special PverOBPs (*OBP33* and *37*) were highly expressed in pupae which were also consistent with the results obtained in other species. For example, five OBPs in *Procecidochares utilis*, *OBP44a* in *Bactrocera dorsalis*, and three OBPs (*OBP15*, *17*, and *25*) in *Galeruca daurica* were found to be abundantly expressed in the pupal stage, respectively [79–81]. In addition, *PverOR42* was highly expressed in pupae, the same as three ORs in *P. utilis* and eight ORs in *Chlorops oryzae* [79, 82]. These results suggest that these OBPs and ORs in *P. versicolora* may be related to the beginning of the development of chemosensory tissue during pupation. On the other side, maybe it is also likely that these ORs and OBPs are a consequence of development.

Different expression profiles during development indicate that these CSP genes might have different functions. *PverCSP12* has a high expression level in the third instar larva stage, which might suggest a role of these proteins in food searching and feeding. Particularly interestingly, *PverCSP13* was highly expressed in the eggs. It has been demonstrated that *CSP5* in the eggs of honeybees is required for the correct development of the embryo [83, 84]. It suggested that *PverCSP13* may involve in the development of the eggs.

## Supplementary Information

**Additional file 1: Table S1.** The classification and number among different species used in OBPs, CSPs and GRs in the phylogenetic trees. OBPs and CSPs in non-coleoptera species were not used in the phylogenetic analyses. The number of GR in non-coleoptera species of annotated proteins in those genomes were marked in green, in the transcriptome data was marked in bule. The italic numbers were used to structure the GR tree. **Table S2.** Primers for qRT-PCR of chemosensory genes in *P. versicolora*. **Table S3.** The Blastx match of *P. versicolora* candidate CSP and OBP genes. The new genes compared to previous reports were marked with bule color. **Table S4.** The Blastx match of *P. versicolora* candidate GR, IR, OR and SNMP genes. The new genes compared to previous reports were marked with bule color. **Fig. S1.** Distribution of unigene size of transcriptome assembly from third instar larvae head in *P. versicolora*. **Fig. S2.** Gene ontology (GO) classification of transcriptome unigenes from third instar larvae head in *P. versicolora*. **Figure S3.** Phylogenetic analysis of the IRs. The *P. versicolora* genes are shown in blue. The values at the branch nodes represent bootstrap values based on 1000 replicates. **Figure S4.** The Venn diagrams of chemosensory genes in *P. versicolora* from the transcriptome of antennae, forelegs and larvae. **Figure S5.** Phylogenetic analysis of the ORs. The *P. versicolora* genes are shown in blue. Pver, *P. versicolora*; Harm, *Helicoverpa armigera*; Cbow, *Colaphellus bowringi*. The values at the branch nodes represent bootstrap values based on 1000 replicates. **File S1.** The amino acid sequences of *Plagiodera versicolora* putative chemosensory genes.

## Data Availability

The raw reads of the transcriptomes in this study have been submitted in the NCBI SRA database, under the accession number of SAMN29260310 (larvae head 1), SAMN29260311 (larvae head 2) and SAMN29260312 (larvae head 3).

## Abbreviations

OBP: Odorant binding protein
CSP: Chemosensory protein
OR: Odorant receptor
GR: Gustatory receptor
IR: Ionotropic receptor
SNMP: Sensory neuron membrane protein
Nr: Non-redundant database
COG: Clusters of Orthologous Groups
KEGG: Kyoto Encyclopedia of Genes and Genome
GO: Gene Ontology
NCBI: National Center for Biotechnology Information
ORF: Open reading frame
TMD: Transmembrane domain
