# Correction: Transcriptome analysis and identification of chemosensory genes in the larvae of *Plagiodera versicolora*

**DOI:** 10.1186/s12864-023-09132-8

**Published:** 2023-05-05

**Authors:** Zhe-Ran Wu, Jian-Ting Fan, Na Tong, Jin-Meng Guo, Yang Li, Min Lu, Xiao-Long Liu

**Affiliations:** 1 State Key Laboratory of Biocatalysis and Enzyme Engineering, School of Life Sciences, Hubei University, Wuhan, 430062 China; 2 School of Forestry and Biotechnology, Zhejiang A & F University, National Joint Local Engineering Laboratory for High-Efficient Preparation of Biopesticide, Lin’an, 311300 China; 3 Key Laboratory of Integrated Management of Crop Disease and Pests, Ministry of Education/ Department of Entomology, Nanjing Agricultural University, Nanjing, 210095 China

**Correction: BMC Genomics 23, 845 (2022)**

**https://doi.org/10.1186/s12864-022-09079-2**

Following the publication of the original article [1], it was noted that the figure legends were paired incorrectly. The figure legends for Figs. 2 and 3 were wrongly given as captions for Figs. 3 and 2 respectively.

**Fig. 2:**
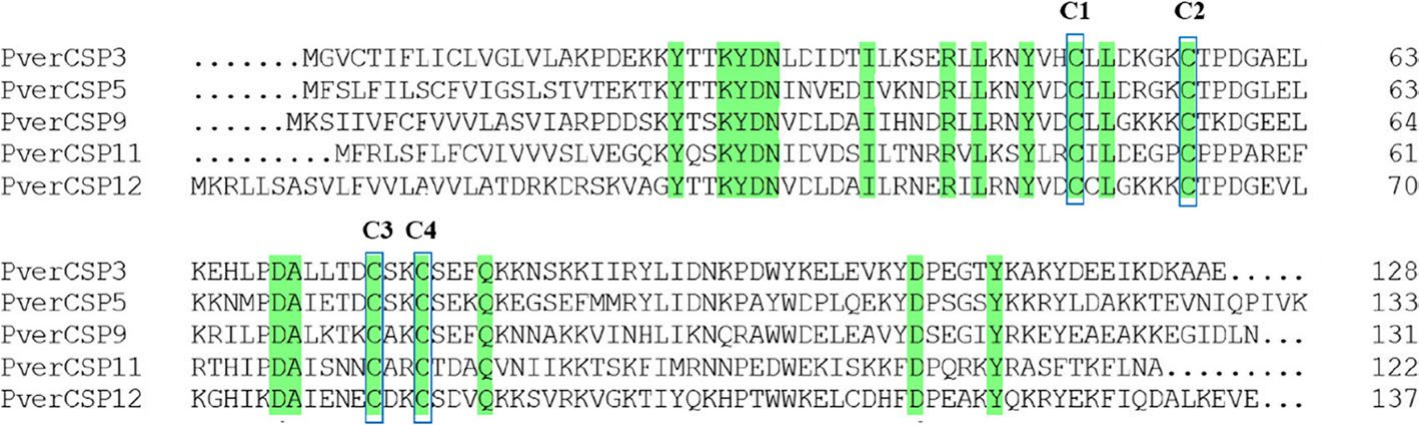
Multiple amino alignments of the predicted CSPs. Conserved cysteine (C1-C4) residues are marked with a blue box

**Fig. 3:**
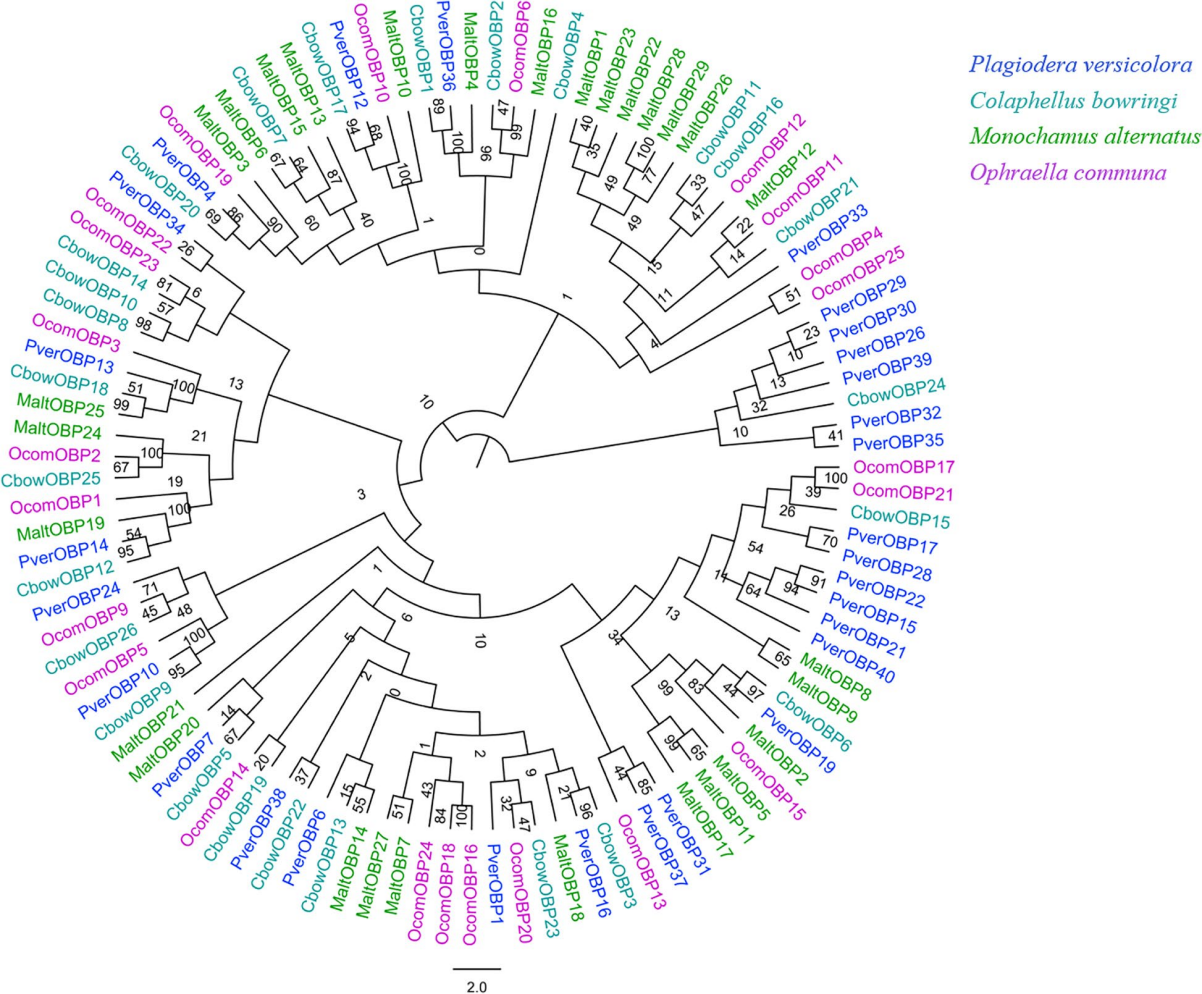
Phylogenetic analysis of the OBPs (odorant-binding proteins) from four insect species: *P. versicolora* (Pver), *C. bowringi* (Cbow), *M. alternatus* (Malt), *O. communa* (Ocom). The *P. versicolora* genes are shown in blue. The values at the branch nodes represent bootstrap values based on 1000 replicates

The correct figures and captions have been included in this correction, and the original article has been corrected.
