# Supplementary material for: Transcriptome analysis and identification of chemosensory genes in the larvae of Plagiodera versicolora
Source: BMC Genomics. 2022 Dec 22;23:845. doi: 10.1186/s12864-022-09079-2 (PMC9773597; doi:10.1186/s12864-022-09079-2)
Supplement: Supplementary file 1 — Additional file 1: Table S1. The classification and number among different species used in OBPs, CSPs and GRs in the phylogenetic trees. OBPs and CSPs in non-coleoptera species were not used in the phylogenetic analyses. The number of GR in non-coleoptera species of annotated proteins in those genomes were marked in green, in the transcriptome data was marked in bule. The italic numbers were used to structure the GR tree. Table S2. Primers for qRT-PCR of chemosensory genes in P. versicolora. Table S3. The Blastx match of P. versicolora candidate CSP and OBP genes. The new genes compared to previous reports were marked with bule color. Table S4. The Blastx match of P. versicolora candidate GR, IR, OR and SNMP genes. The new genes compared to previous reports were marked with bule color. Fig. S1. Distribution of unigene size of transcriptome assembly from third instar larvae head in P. versicolora. Fig. S2. Gene ontology (GO) classification of transcriptome unigenes from third instar larvae head in P. versicolora. Figure S3. Phylogenetic analysis of the IRs. The P. versicolora genes are shown in blue. The values at the branch nodes represent bootstrap values based on 1000 replicates. Figure S4. The Venn diagrams of chemosensory genes in P. versicolora from the transcriptome of antennae, forelegs and larvae. Figure S5. Phylogenetic analysis of the ORs. The P. versicolora genes are shown in blue. Pver, P. versicolora; Harm, Helicoverpa armigera; Cbow, Colaphellus bowringi. The values at the branch nodes represent bootstrap values based on 1000 replicates. File S1. The amino acid sequences of Plagiodera versicolora putative chemosensory genes. [file 12864_2022_9079_MOESM1_ESM.docx]

**Supplementary data**

**Table S1.** The classification and number among different species used in OBPs, CSPs and GRs in the phylogenetic trees. OBPs and CSPs in non-coleoptera species were not used in the phylogenetic analyses. The number of GR in non-coleoptera species of annotated proteins in those genomes were marked in green, in the transcriptome data was marked in bule. The italic numbers were used to structure the GR tree.

| Order | Family | Species | OBPs | CSPs | GRs |
| --- | --- | --- | --- | --- | --- |
| Coleoptera | Chrysomelidae | *Plagiodera versicolora* | 29 | 6 | *13* |
|  | Chrysomelidae | *Colaphellus bowringi* | 26 | 12 | *10* |
|  | Cerambycidae | *Monochamus alternatus* | 29 | 12 | - |
|  | Chrysomelidae | *Ophraella communa* | 25 | 11 | *17* |
|  | Colydiidae | *Dastarcus helophoroides* | - | 7 | - |
| Lepidoptera | Plutellidae | *Plutella xylostella* | - | - | 69 (*2*) |
|  | Noctuidae | *Helicoverpa armigera* | - | - | 213 (*5*) |
|  | Bombycidae | *Bombyx mori* | - | - | 69 (*3*) |
| Diptera | Culicidae | *Anopheles gambiae* | - | - | 59 (*3*) |
|  | Culicidae | *Aedes aegypti* | - | - | 91 (*3*) |
|  | Culicidae | *Anopheles coluzzii* | - | - | 23 (*3*) |
|  | Drosophilidae | *Drosophila melanogaster* | - | - | *60* |

**Table S2.** Primers for qRT-PCR of chemosensory genes in *P. versicolora*.

| OBP31-F | TGTACCTCAGGCTCGGTATAA | GR22-F | TGGAGAAACTGCGTGCTAAA |
| --- | --- | --- | --- |
| OBP31-R | ACCTATGAACCGCCCTACT | GR22-R | GTTTGACAGATCGCACCAAATAC |
| OBP33-F | AGTTCTGAAAGAGGGCAAAGG | GR24-F | CTTCTCCTTCGGCTTTCTAGTC |
| OBP33-R | CACTGCTTCATAGCCCAGAAT | GR24-R | CCAAAGGCTTCCTTGTTTCTTC |
| OBP34-F | CTCCCAAGGCAACGAAAGA | CSP12-F | CTCCTGACGGCGAAGTATTG |
| OBP34-R | CAGGAGCGAATTTGACAAACG | CSP12-R | TACCCACTTTGCGTACTGATTT |
| OBP36-F | CTCAAGTCAGTTGCTCCAGAA | CSP13-F | TGAAGCTCTGCCGGAAATC |
| OBP36-R | CAATCTCCAAAGCTCCCATACA | CSP13-R | CTGGACCAAAGCATTCCAAAC |
| OBP37-F | ATCGGTATCGGTTCTTGTTTGT | IR12-F | CCTTAAGCACTGAAGATTTCTTTGA |
| OBP37-R | CCTTGTCCATCAGGTCTTCTTC | IR12-R | GCCTACTTGATGGAGCCTTT |
| OBP38-F | CAGTGTGTGTACAAGGAGACAG | IR14-F | GACTAGTTACCAGCATGGAAGG |
| OBP38-R | ATCCGGGTAGAACTGGGATAA | IR14-R | GATACAAATGGCGGTTGATGTG |
| OBP39-F | ACCGTTGGTCTCAAGAACTG | OR41-F | CTATGGGAGCTGTGCAGAAA |
| OBP39-R | GCACACAGAATGCTGCAATAG | OR41-R | CAGAGTAGCATGGGCTAAGATG |
| OBP40-F | ATTGTTGACGAGTGTGGAGAG | OR42-F | CATGTCCTACAACTTGCTGAAAC |
| OBP40-R | GCAGGACGGTGTTTCCTATAA | OR42-R | GTGTTCATTGCAGCCCAATAC |
| q18S-F | CTTCCTCGTCGGAGCATTCT | q18S-R | GTTCGCCTTAACTGCCATCAA |

**Table S3**. The Blastx match of *P. versicolora* candidate CSP and OBP genes. The new genes compared to previous reports were marked with bule color.

| Name | ORF  (aa) | Signal peptide | complete | Blast best hit  ACC. NO. Gene Species | Evalue | Identity  (%) |
| --- | --- | --- | --- | --- | --- | --- |
| **CSP** | | | | | | |
| CSP3 | 128 | 1-17 | Y | QFO46789.1\| chemosensory protein [Cylas formicarius] | 5e-56 | 68.8 |
| CSP5 | 134 | 1-16 | Y | ALR72526.1\| chemosensory protein 12 [Colaphellus bowringi] | 1e-68 | 80.7 |
| CSP9 | 131 | 1-18 | Y | ALR72517.1\| chemosensory protein 3 [Colaphellus bowringi] | 5e-48 | 64.9 |
| CSP11 | 122 | 1-19 | Y | AUF73000.1\| chemosensory protein [Anoplophora chinensis] | 8e-48 | 65.4 |
| CSP12 | 137 | 1-19 | Y | ALR72516.1\| chemosensory protein 2 [Colaphellus bowringi] | 3e-51 | 63.5 |
| CSP13 | 79 | N | N | ALR72522.1\| chemosensory protein 8 [Colaphellus bowringi] | 1e-19 | 77.5 |
| **OBP** | | | | | | |
| OBP1 | 131 | 1-19 | Y | AIX97052.1\| odorant-binding protein 6 [Dastarcus helophoroides] | 4e-25 | 40.3 |
| OBP4 | 132 | 1-17 | Y | ALR72508.1\| odorant binding protein 20 [Colaphellus bowringi] | 9e-56 | 60.6 |
| OBP6 | 61 | N | N | AWK23450.1\| odorant-binding protein 13 [Chrysomela populi] | 5e-27 | 90.9 |
| OBP7 | 131 | 1-17 | Y | AXO78397.1\| odorant binding protein 19 [Xylotrechus quadripes] | 3e-21 | 43.6 |
| OBP10 | 155 | 1-20 | Y | ALR72497.1\| odorant binding protein 9 [Colaphellus bowringi] | 2e-39 | 65 |
| OBP12 | 136 | 1-18 | Y | ALR72505.1\| odorant binding protein 17 [Colaphellus bowringi] | 8e-70 | 75 |
| OBP13 | 183 | 1-23 | Y | AUF72969.1\| odorant-binding protein [Anoplophora chinensis] | 3e-84 | 70 |
| OBP14 | 241 | 1-18 | Y | ALR72500.1\| odorant binding protein 12 [Colaphellus bowringi] | 1e-61 | 47.2 |
| OBP15 | 129 | 1-19 | Y | AQY18986.1\| odorant-binding protein [Galeruca daurica] | 5e-26 | 40.6 |
| OBP16 | 134 | 1-17 | Y | AXO78395.1\| odorant binding protein 17 [Xylotrechus quadripes] | 2e-41 | 47.7 |
| OBP17 | 143 | 1-27 | Y | ALR72503.1\| odorant binding protein 15 [Colaphellus bowringi] | 2e-16 | 40.4 |
| OBP19 | 135 | 1-19 | Y | ALR72494.1\| odorant binding protein [Colaphellus bowringi] | 4e-46 | 65.2 |
| OBP21 | 138 | 1-19 | Y | AQY18990.1\| odorant-binding protein [Galeruca daurica] | 1e-21 | 40.8 |
| OBP22 | 133 | 1-19 | Y | ALR72503.1\| odorant binding protein 15 [Colaphellus bowringi] | 3e-25 | 55.2 |
| OBP24 | 148 | 1-16 | Y | AWT23276.1\| OBP5 [Hycleus cichorii] | 9e-30 | 37.6 |
| OBP26 | 92 | N | N | ALR72494.1\| odorant binding protein [Colaphellus bowringi] | 5e-35 | 70.4 |
| OBP28 | 134 | 1-23 | Y | ALR72503.1\| odorant binding protein 15 [Colaphellus bowringi] | 6e-24 | 40.91 |
| OBP29 | 105 | N | N | ALR72513.1\| odorant binding protein 25 [Colaphellus bowringi] | 3e-35 | 69.1 |
| OBP30 | 93 | N | N | ALR72513.1\| odorant binding protein 25 [Colaphellus bowringi] | 3e-30 | 70.2 |
| OBP31 | 146 | 1-20 | Y | AIX97023.1\| odorant-binding protein 8 [Monochamus alternatus] | 6e-12 | 31.3 |
| OBP32 | 63 | N | N | ALR72495.1\| odorant binding protein 7 [Colaphellus bowringi] | 2e-23 | 77.8 |
| OBP33 | 145 | 1-17 | Y | APC94284.1\| odorant-binding protein 30 [Pyrrhalta aenescens] | 8e-10 | 32.7 |
| OBP34 | 123 | 1-25 | Y | ARH65468.1\| odorant binding protein 13 [Anoplophora glabripennis] | 7e-10 | 38.3 |
| OBP35 | 107 | N | N | AQY18990.1\| odorant-binding protein [Galeruca daurica] | 8e-16 | 39.2 |
| OBP36 | 142 | 1-20 | Y | ALR72489.1\| odorant binding protein 1 [Colaphellus bowringi] | 3e-61 | 62.7 |
| OBP37 | 132 | 1-19 | Y | AUF72972.1\| odorant-binding protein [Anoplophora chinensis] | 1e-15 | 36.6 |
| OBP38 | 259 | 1-20 | Y | QKV34985.1\| Odorant binding protein 4 [Dendroctonus adjunctus] | 1e-18 | 28.3 |
| OBP39 | 171 | N | N | ALR72504.1\| odorant binding protein 16 [Colaphellus bowringi] | 3e-24 | 36.0 |
| OBP40 | 160 | 1-23 | Y | APC94279.1\| odorant-binding protein 4 [Pyrrhalta aenescens] | 1e-21 | 42.6 |

**Table S4.** The Blastx match of *P. versicolora* candidate GR, IR, OR and SNMP genes. The new genes compared to previous reports were marked with bule color.

| Name | ORF  (aa) | TMD | complete | Blast best hit  ACC. NO. Gene Species | Evalue | Identity  (%) |
| --- | --- | --- | --- | --- | --- | --- |
| **GR** | | | | | | |
| GR3 | 86 | – | N | AUF73052.1\| gustatory receptor [Anoplophora chinensis] | 3e-19 | 50.0 |
| GR7 | 101 | – | N | APC94333.1\| gustatory receptor 3 [Pyrrhalta aenescens] | 2e-28 | 28.7 |
| GR9 | 111 | – | N | AVN97874.1\| gustatory receptor 9 [Anoplophora chinensis] | 4e-80 | 38.2 |
| GR11 | 131 | – | N | AWK23449.1\| gustatory receptor 1 [Chrysomela populi] | 3e-67 | 79.4 |
| GR12 | 289 | – | N | AVN97874.1\| gustatory receptor 9 [Anoplophora chinensis] | 4e-70 | 41.8 |
| GR15 | 345 | 7 | Y | RZC42344.1\| gustatory and odorant receptor 24  [Asbolus verrucosus] | 9e-153 | 65.5 |
| GR18 | 130 | – | N | RZC41799.1\| gustatory receptor 2 [Asbolus verrucosus] | 5e-53 | 70.9 |
| GR19 | 147 | – | N | APC94332.1\| gustatory receptor 2 [Pyrrhalta aenescens] | 2e-28 | 40.0 |
| GR20 | 126 | – | N | AVN97870.1\| gustatory receptor 5 [Anoplophora chinensis] | 4e-18 | 44.3 |
| GR21 | 215 | – | N | AUF73059.1\| gustatory receptor [Anoplophora chinensis] | 3e-30 | 54.5 |
| GR22 | 102 | – | N | AUF73051.1\| gustatory receptor [Anoplophora chinensis] | 2e-09 | 44.2 |
| GR23 | 302 | – | N | KYB27621.1\| Gustatory receptor for sugar taste 64f-like Protein [Tribolium castaneum] | 5e-106 | 51.8 |
| GR24 | 67 | – | N | AVN97870.1\| gustatory receptor 5 [Anoplophora chinensis] | 3e-21 | 55.2 |
| **IR** | | | | | | |
| IR2 | 639 | 3 | Y | AKC58589.1\| ionotropic receptor 75q [Anomala corpulenta] | 2e-95 | 33.8 |
| IR5 | 926 | 3 | Y | ALR72535.1\| ionotropic receptor IR6 [Colaphellus bowringi] | 0.0 | 83.5 |
| IR9 | 917 | 4 | Y | ANQ46493.1\| ionotropic receptor 1 [Phyllotreta striolata] | 0.0 | 66.9 |
| IR10 | 81 | – | N | AJO62241.1\| chemosensory ionotropic receptor IR3  [Tenebrio molitor] | 5e-42 | 89.6 |
| IR11 | 74 | – | N | AIX97122.1\| ionotropic receptor 6 [Rhyzopertha dominica] | 6e-34 | 77.0 |
| IR12 | 126 | – | N | AUF73071.1\| ionotropic receptor [Anoplophora chinensis] | 1e-38 | 65.1 |
| IR13 | 64 | – | N | QBB73021.1\| ionotropic receptor [Protaetia brevitarsis] | 1e-18 | 60.0 |
| IR14 | 322 | – | N | APC94262.1\| ionotropic receptor 4 [Pyrrhalta maculicollis] | 1e-88 | 50.7 |
| **OR** | | | | | | |
| OR3 | 45 | – | N | AUF73019.1\| odorant receptor [Anoplophora chinensis] | 2e-15 | 71.1 |
| OR9 | 143 | – | N | ALR72565.1\| odorant receptor OR20 [Colaphellus bowringi] | 5e-18 | 45.8 |
| OR26 | 69 | – | N | ALR72583.1\| odorant receptor OR40 [Colaphellus bowringi] | 1e-31 | 82.6 |
| OR27 | 188 | – | N | AUF73039.1\| odorant receptor [Anoplophora chinensis] | 3e-21 | 34.8 |
| OR36 | 200 | – | N | QXE93269.1\| odorant receptor 43 [Eucryptorrhynchus brandti] | 4e-36 | 34.3 |
| OR38 | 48 | – | N | QXE93221.1\| odorant receptor 41 [Eucryptorrhynchus scrobiculatus] | 1e-73 | 31.3 |
| OR39 | 49 | – | N | AVN97831.1\| odorant receptor 19 [Anoplophora chinensis] | 4e-07 | 55.5 |
| Orco | 289 | – | N | QEE83332.1\| odorant co-receptor [Ophraella communa] | 4e-179 | 94.4 |
| OR40 | 220 | – | N | AVN97822.1\| odorant receptor 10 [Anoplophora chinensis] | 8e-76 | 52.7 |
| OR41 | 316 | – | N | ALR72556.1\| odorant receptor OR11 [Colaphellus bowringi] | 1e-173 | 73.4 |
| OR42 | 239 | – | N | ALR72564.1\| odorant receptor OR19 [Colaphellus bowringi] | 3e-67 | 44.8 |
| OR43 | 86 | – | N | EEZ99426.1\| odorant receptor 322 [Tribolium castaneum] | 0.03 | 32.2 |
| OR44 | 92 | – | N | ALR72565.1\| odorant receptor OR20 [Colaphellus bowringi] | 5e-18 | 45.8 |
| OR45 | 45 | – | N | AVN97859.1\| odorant receptor 47 [Anoplophora chinensis] | 6e-13 | 72.7 |
| **SNMP** | | | | | | |
| SNMP1a | 482 |  | Y | ALR72542.1\| sensory neuron membrane protein SNMP1a [Colaphellus bowringi] | 0 | 66.0 |
| SNMP1b | 522 |  | Y | ALR72543.1\| sensory neuron membrane protein SNMP1b [Colaphellus bowringi] | 0 | 54.9 |
| SNMP2a | 515 |  | Y | ALR72544.1\| sensory neuron membrane protein SNMP2 [Colaphellus bowringi] | 0 | 57.4 |
| SNMP2b | 506 |  | Y | ALR72545.1\| sensory neuron membrane protein SNMP3 [Colaphellus bowringi] | 0 | 63.6 |

**Fig. S1.** Distribution of unigene size of transcriptome assembly from third instar larvae head in *P. versicolora*.


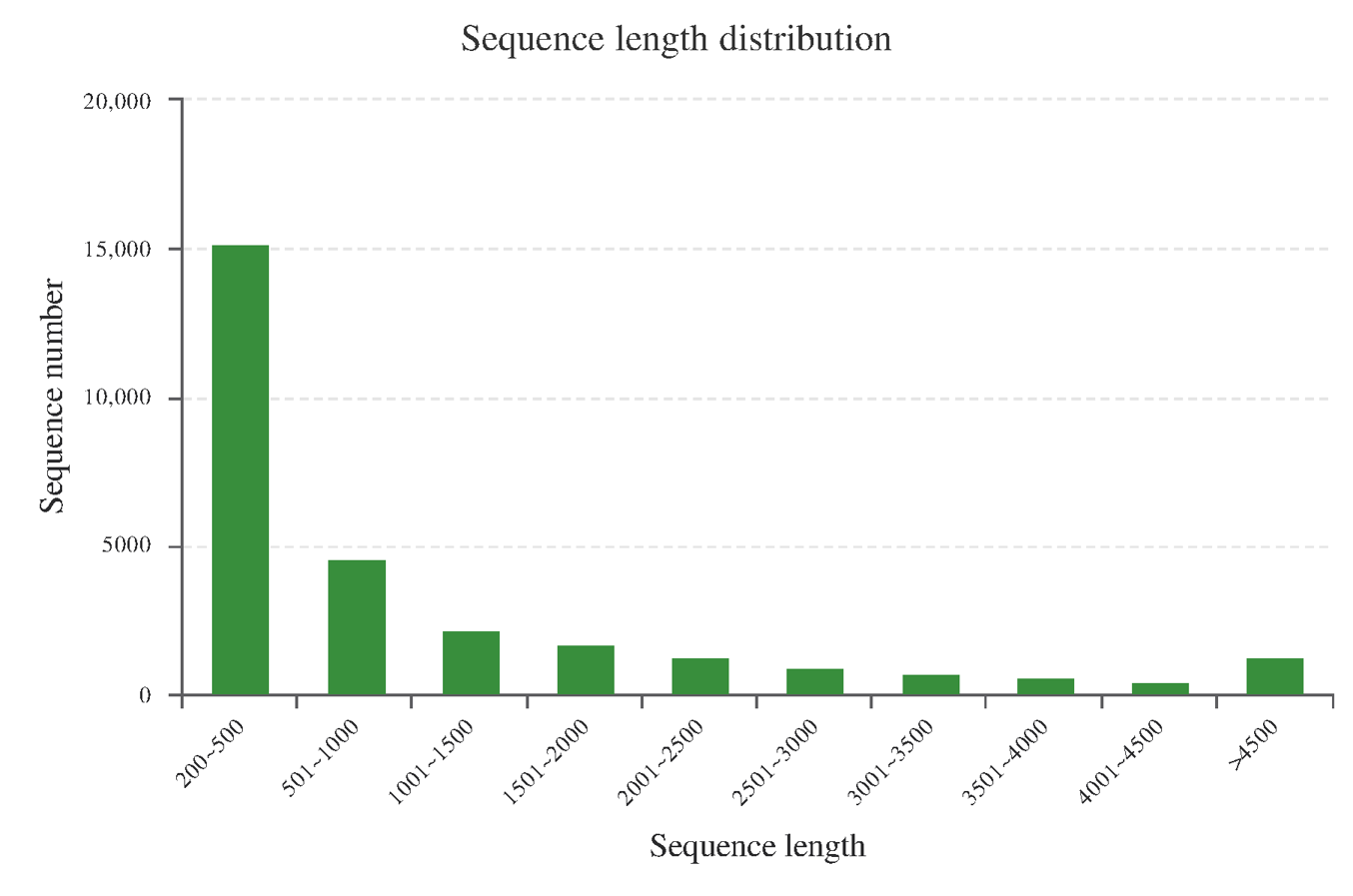


**Fig. S2.** Gene ontology (GO) classification of transcriptome unigenes from third instar larvae head in *P. versicolora*.


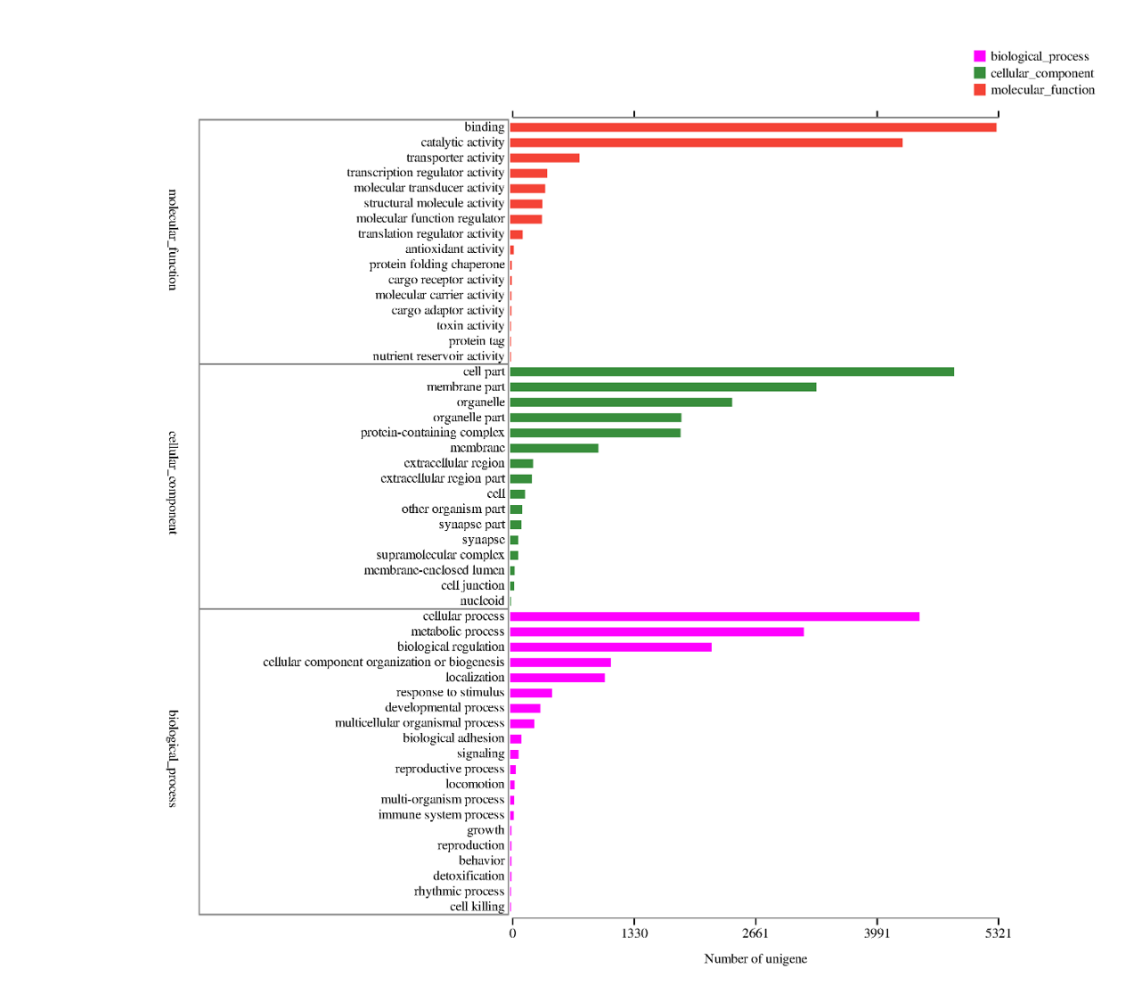


**Figure S3.** Phylogenetic analysis of the IRs. The *P. versicolora* genes are shown in blue. The values at the branch nodes represent bootstrap values based on 1000 replicates.
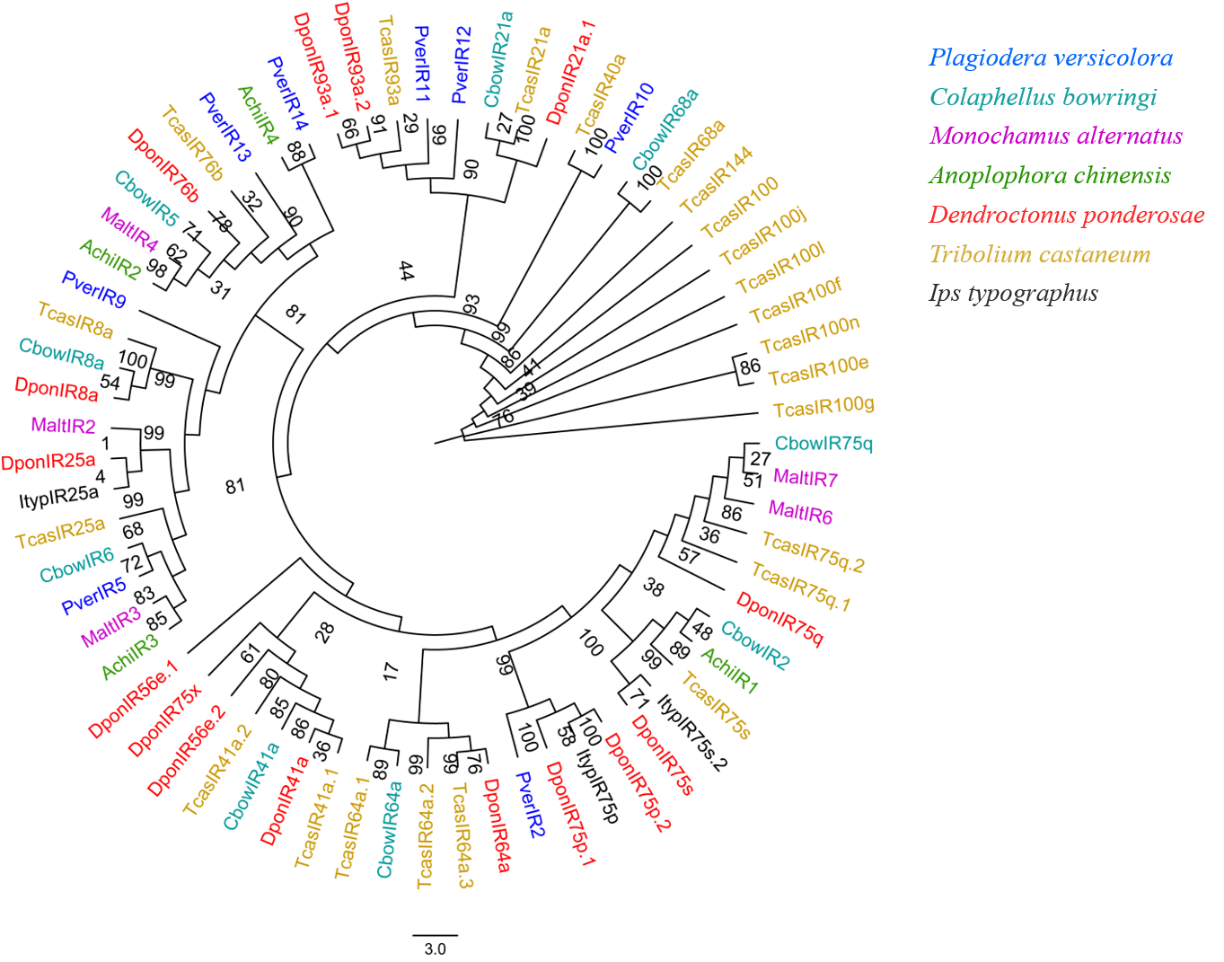


**Figure S4.** The Venn diagrams of chemosensory genes in *P. versicolora* from the transcriptome of antennae, forelegs and larvae.

**
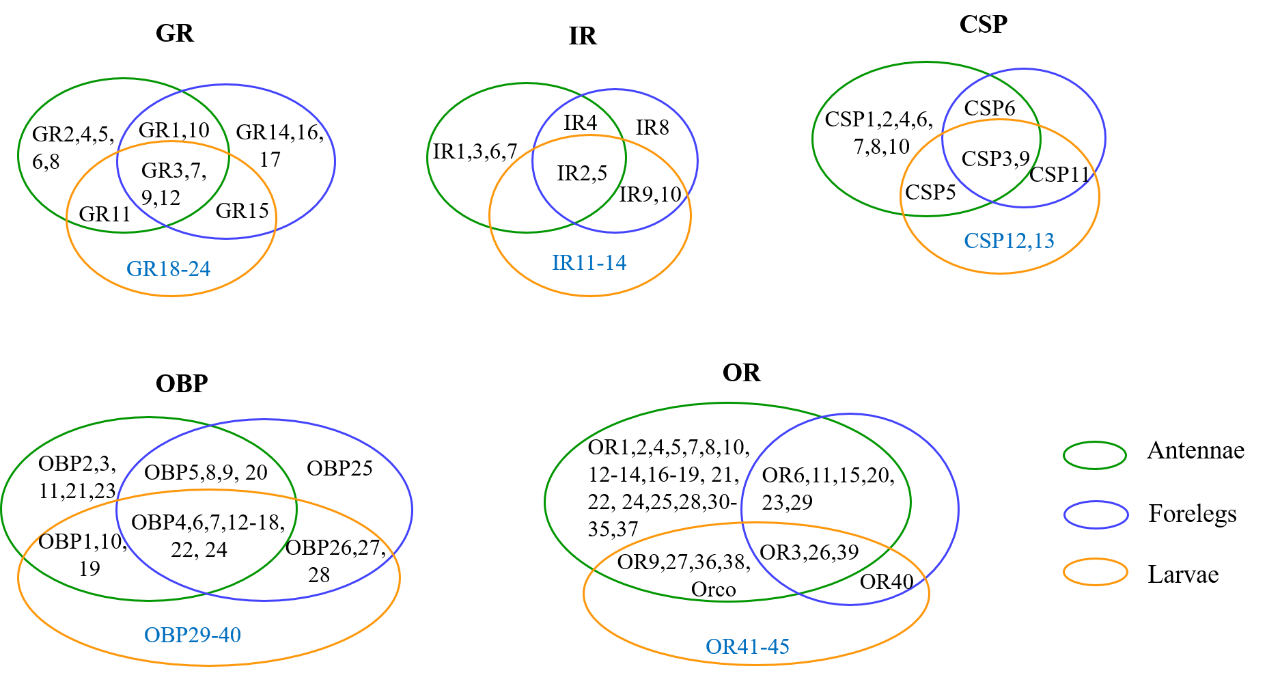
**

**Figure S5**. Phylogenetic analysis of the ORs. The *P. versicolora* genes are shown in blue. Pver, *P. versicolora*; Harm, *Helicoverpa armigera*; Cbow, *Colaphellus bowringi*. The values at the branch nodes represent bootstrap values based on 1000 replicates.

**
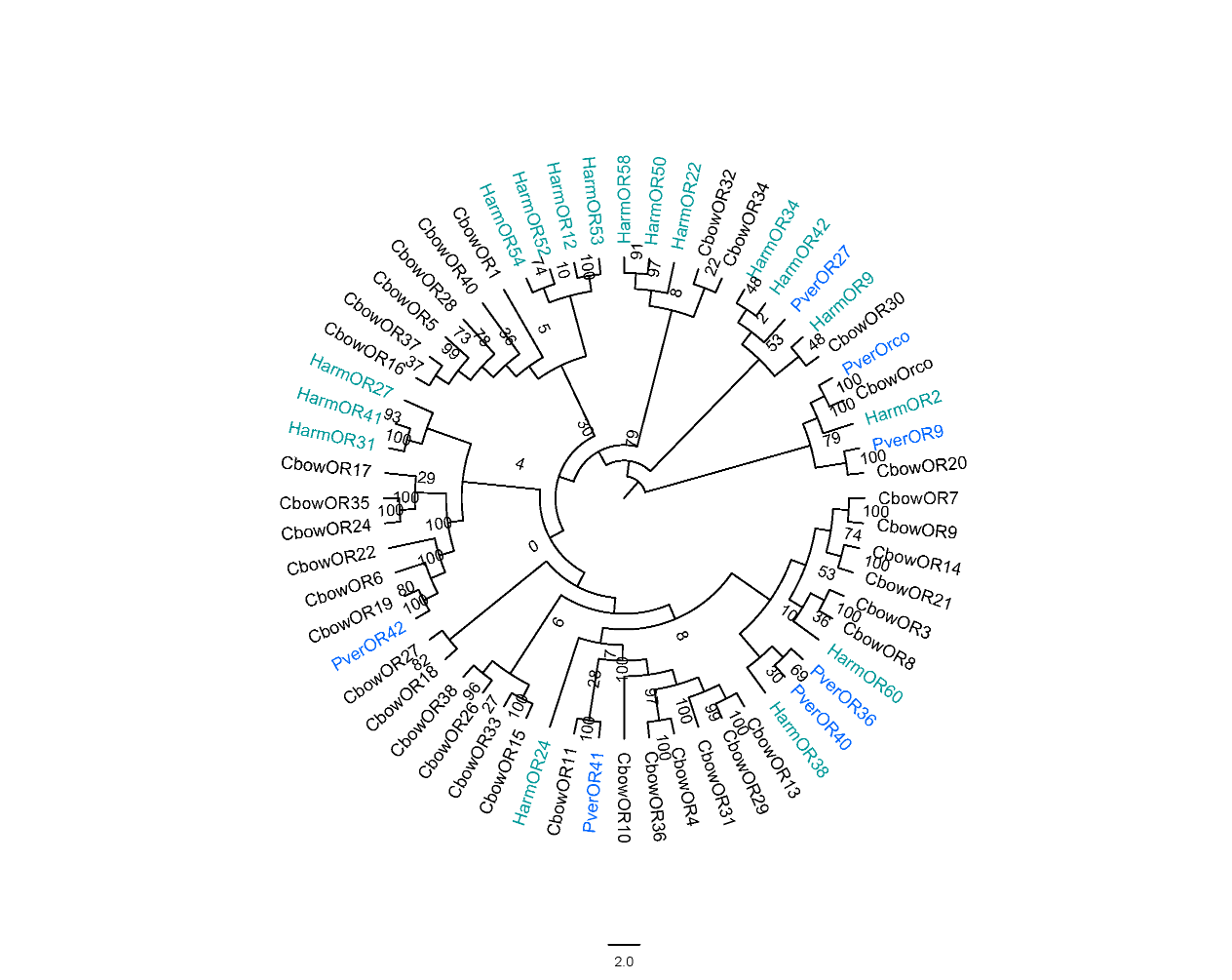
**

**File S1.** The amino acid sequences of *Plagiodera versicolora* putative chemosensory genes.

**OBP**

>PverOBP1

MKIIVLVCFAFAFFENGKCFSDEEKTMIMNIHTECVGMSGITDAMIEQARTGDFPPDSEFKEYLLCFAKKAGVMNESGELQTGKITKMVQMHIADAAKADKMVETCLVNKETPQDTIFETAKCMFEVYRFF

>PverOBP4

MFSFIVVGLCIICSISAFTEEEQQMMEALHAECVSQTGCPEDLISRASSGDFPEDEKLKCYMKCIFNELGVIDDDGKIDSSGLVAMFPEDIQAIAKPIFAKCGTVAGTDLCDSIYQTNKCYYGENPGAYFLP

>PverOBP6

MQSSGITPELLAKTKKGEFPDNQKLKEHMYCFAKKAELMDAQGKIKKDVLLAKVSAIESFE

>PverOBP7

MGKVLFALFLVFTVAFCKPLSDEVKAEILKIHEECAVTSGVNPREILDKVFGEGGDEDQIKTHVFCIGQKLKVIDDDNKIDRETLKTHLSEIITEDGEVEEIVTKCAVEKDDAKETAYYLTKCIHESMQKE

>PverOBP10

MKSTYFTLLLATSLFSATLGARRGRTGLLDPSNHKKVLNDCRTQSGATNSDMEAIKMKKLPDTKTGRCLVQCIFNNAKIMDEGKFNKNGMVIAFTPALKGDLTKLGKLKQLSEVCDKEIGPAVSKDCEGPKKIVECIAKHGSAYGFSYDNTNVNL

>PverOBP12

MTLKSFLYFTVLLGLGSCIELPPELQEFVEDLHKICVSKSGITESDYAAYDVKGNPHDTKLQCYMKCLMMEAKWMNSAGAIQYDFIIDTAHPTIKDLLEPAINKCRKIDEGANLCEKASNFNFCMYEADPENWYLI

>PverOBP13

MSTVRHCLLYGVCFLVFNVRIEAAEIHKNYTNKCDIPPTAPKKIEAVINQCQDEIKLAILSEALESLNVNEHTHSRAKRAAFSDDERRIAGCLLQCVYKKMNAINDKGFPTADGLVSLYTDGISQKDYILATVDAVKYCLSFAQKKFRVTPNSIEVHGMSCDIAYDVFDCVSDEIANYCGQSP

>PverOBP14

MRGLILIFVSCYVVHSKAIECGIDKSNRDEIKQALAMCVKNNATLNKIWEMTSSAQTTPSSTEEGTDSMEENDKSVPSIPTRNIAKNQRSGKSGRIKRAKSMKSFNTQKFSTTTMRSSDDSNGMKNSDEEEKNESNDVSEDNENSRQDSTDKCIIHCVLEKMSLTDDNGLPDHSKILEELLKNTPKRELKNFLQDSTDECFQEVDEANESDSCEYSNKLIFCLAEKGRSNCADWPAGSLPF

>PverOBP15

MKPIALVAFILVLVAMIAAEDIHSRMKNIHEECQADPATKIDHGVMEKFFEGEKVDQDQFARHSLCMNKKIGLQKENGDVDKDALRKIFEHNDKLDEAVEECGQGKGTAEETALALLKCIKKYRPSRTP

>PverOBP16

MNSVLVLVICLISIVKAGMLTDEQKEKILKYGQECLKESNVDFDVVMDAAKGKYADDPNLKKQILCFNKKIGVQDSDGKLVMDTVKARLMGITNDPKKTEDIIKECVIEKSTPEDTAFETAKCLHRLAPDEKIV

>PverOBP17

MFSIRTMKIFLVTICVVSFVVMALVSGKDLRIKTYFHECQSDPKTYVDEELLQKGLKGELADETPVGPHAFCMNLKRGFQVPNGDVDVVALRTYLEQSESRNDTLIDRAIKECGQRNGSTAQQAALALLDCIHRIIPHKDENH

>PverOBP19

MKSTLVVFSCIVVAVLANSLPESERLKLARVHAECQANPKTRCDENLLRNLGANANNAQVGIHMLCMSVKEGLQRPNGDLDRNFIKSKIDLVSDDKSKVDHYLQTCAVKKQTPEKTAVDLVLCFVQNGIPYYYRL

>PverOBP21

MKSIALIVFILAFTAMIDADDIQDEREKARKVHGECQADPATKIDADAMDRFFRDEAVNEAQLAKHSLCMNKKMGLQNENGDIDKDLMRKLIRHDSNVDEIVAECGQRKGSAEETTLALLKCMKKYRHREDEHHHHGH

>PverOBP22

MKSIALIAFILALGAMIAADDIQERVEKMKKIHGECQADPATKVDEEDMEKFFRSETIDEAQFAKHTLCMNKKLGLQKENGDVDKDNLRKSAERSDKVDEIVEECGQRKGTAEETALAVLKCIRKYLPKQEHH

>PverOBP24

MQRLIVFAALTIAVQSVDQSFINEFKEKATEIGIKCVEQTGASNDDIASIMDQKVPSSKEGKCMISCFHKAVGVQNDDGSLNPNGMKVFMEKLKSNDEDMYNKFRQLLDECLIPGNVLPDHCDTSAKVATCAIEVAKKVGLSSSFMKL

>PverOBP26

MRMISQSIIIGNSILDKTKHQINSSFLRSLLFHSACLQIMVYFRLIVRYQVDFALDEVSVQVSVGSLQTLFHRHAEHVNSNLSVVGVGSEVS

>PverOBP28

MNTMGNITVIACILAFMTLSVSCRNDRLRRFHTECQLDPAYRVEEGLIRKAMRGETVDESVVGPHVLCINIKAGIQNQNGNVIEKGLRTILKERIKDDSVVEDVMSRCGHKNGGTPHEAAVALTRCLHKHARNH

>PverOBP29

MTNAFRKPPIITHKHILSELFHFLTVEWIKWTSMVFIWSSLRFVLACYLLETSSLYVFVVIIWLPEQFFATAQRFSSSRATFILFRSGFFSTYSRMIESVSVDVF

>PverOBP30

MHSENLLSLLTSISFLNCSTFSQLNGSNGPPWCLSGLPSGLSLPVISLKQALFMSLSSSFGFLNSSSPQHRGFPLLGRHLSSSEVVSSLRTLG

>PverOBP31

MKACTVFFCIVSALITLVNTQSNLFGPFLDAHEACQENPETRIVGNLMDMAAAGQPVDEVSFGNHMLCMYLRLGIMSRDGRVNASRTIDLLTDVLNDRPVVSRAVHRCCGPRRPFQTPQQVASELFKCVTRNLPGLSTVKPPASEP

>PverOBP32

MILNHTHISCFMLITFVCQPCILTRGGTFDCTTFSYYRCTFGSIFFWENSDSSFFIYNAIIIN

>PverOBP33

MMFSILLIIIASASIQGHMDESEYGCAFSRLVKFLRPYCINKFPITDEDWDIVKEGNFGEHNEKLKKYYECCMTESGLVDGSFEVNEILLKTFMPKVLKEGKGGELIPKCAAKAKAMSDSIPSEDRFWAMKQCIYHGDPENVIIL

>PverOBP34

MKRNMMLNFAGIMFVLSTGLPIILADMNSINEECLKSSGADPKDFEQMPPKATKEVLCYMKCVMDKDKILDGNGNIDVDSFVKFAPDDVNVTILKEVKQCVSKVGKISKCEDIGKIMACLPKK

>PverOBP35

MHLLKATASSSGLPGDLFPHACAKVANRSSSGSETNSFTRSWKSGGVFPSWSWRPIPICMHSRWLPRDGSWTSSPWSNLESSAGSMWVEGLAWHEWWILTNFWLGVS

>PverOBP36

MMKWLIVLINILVIAHYGYSEMSEKQLKATQKLVRNTCQNKVKASSDELDAMRAGNFEQGKTAQCYMLCIMNTYKLFSKEGSFDWQTGVQTLKSVAPEKIAGPGVVSIQNCKDAKKATDKCMGALEIAKCIYNDNPQNYFLP

>PverOBP37

MNSALVFLSVSVLVCLSTAQKNLTGEFRKAHEFCQIRPETRIEEDLMDKAAAGQSVDEYKLGNHLFCMYIKMGIMSRDGKIKKDVLKQQLVEPVKDEAVINRIVRNCATERGTSQETAAELFRCVRRNLTTV

>PverOBP38

MNFFLAVVTTLLVAIHEIPANPVSPNEVRVPLFSHSDCREKSGVDEYHILNPHKALQSEDLKFKEYLLCFLQKSGYVNELGEFQHEPIRKQLNDLYEDQSVVEVVLKECVVKENIPLEAALGFYKCYHRYYPQEGFLYKNSFTDNEKEKLSQIHAKCREESSFEDSLMEQFKAGNFDHPLGKKHLQCVYKETGLFDESGNFNQEKIREKLSQFYPDTNLVQQYLNKCLIKKDSSEETTYYLQSCLYENTPHKFNVFSVL

>PverOBP39

MIGKSQSHRLGIHYVQNMIKSSELILYIHFSISYIFSTKGVTPSTSFQQSDIILGILSFRAGGQSFITISILIIPLVSRTVDSCHKHVIYSLISLFSMKFPCLMLSIAAFCVPVLLIHVLCREVIRSTKSFPKSSIFHVDECPVKDMIKTMYNNFLLFIIYNSLMQFLRNI

>PverOBP40

MSEIMKPIILISSILALVAVVTAKPNAVIPEIEQYRSIQDECRADPATRVDNETIEKFFEGESIDEVKFGKFILCMNIKVRVQNENGDIDKEALKKEMKKVAGENNEMVQEIVDECGEKKGNTALEAALALAKCYRKHRPAFIHEIEHKHGQEQHHQIEK

**CSP**

>PverCSP3

MGVCTIFLICLVGLVLAKPDEKKYTTKYDNLDIDTILKSERLLKNYVHCLLDKGKCTPDGAELKEHLPDALLTDCSKCSEFQKKNSKKIIRYLIDNKPDWYKELEVKYDPEGTYKAKYDEEIKDKAAE

>PverCSP5

MFSLFILSCFVIGSLSTVTEKTKYTTKYDNINVEDIVKNDRLLKNYVDCLLDRGKCTPDGLELKKNMPDAIETDCSKCSEKQKEGSEFMMRYLIDNKPAYWDPLQEKYDPSGSYKKRYLDAKKTEVNIQPIVKS

>PverCSP9

MKSIIVFCFVVVLASVIARPDDSKYTSKYDNVDLDAIIHNDRLLRNYVDCLLGKKKCTKDGEELKRILPDALKTKCAKCSEFQKNNAKKVINHLIKNQRAWWDELEAVYDSEGIYRKEYEAEAKKEGIDLN

>PverCSP11

MFRLSFLFCVLVVVSLVEGQKYQSKYDNIDVDSILTNRRVLKSYLRCILDEGPCPPPAREFRTHIPDAISNNCARCTDAQVNIIKKTSKFIMRNNPEDWEKISKKFDPQRKYRASFTKFLNA

>PverCSP12

MKRLLSASVLFVVLAVVLATDRKDRSKVAGYTTKYDNVDLDAILRNERILRNYVDCCLGKKKCTPDGEVLKGHIKDAIENECDKCSDVQKKSVRKVGKTIYQKHPTWWKELCDHFDPEAKYQKRYEKFIQDALKEVE

>PverCSP13

MVALLGITQENMNIRIQYLYLRFRYISKDHEALPEIIGNNCRSCDNRQLSNARRIAVLVQSKYPDVWNALVQKYSSQSS

**OR**

>PverOR3

MIITMTRMQRPVYLTIGKFTPLTLTALITVFRGSFSYFTVFKSIQ

>PverOR9

MSIKQWILMRNWNAEEKFLLIAGWFPVDLTKHFNLAYFHQISVVFNLTLYSLALDSLIISLVNFAAVRLVILGYKFEHLGSDVGSRTQPVYDSLRDIVMEHKRVIRYVENLNLSLKWYFFGDFVSRSYHLSLTIMNVIRHRSQ

>PverOR26

MVLVEIITALLCMNMYVLSLPNTTVVDYIRCGTMVCAFTTEFLFLYGVPAQKLMDEAEQVANSAFYHCD

>PverOR27

MTENHPIYDSFKYNVLIMKLCAIYPPDSWSNSLYKIYAYSSYITITVLFPILAAIYLFSAEIVDVHQTCWNIILTIETGALIVKVFVFVIKPQQVKKSREDLGKDIFILYAPSQEWIIKDTIQEYYTVFIAITIFSVFSFLAHIFLTAVSLERKFPVNIWLPFDGLTDTKIYVMVFIYITFGKLIVSI

>PverOR36

MIAATTCNNVTQTFFVTMMNFMIGRLIVLQQHFRNFDSYRFDGATITDEKAVLGALKELIKDHLGIIGLVENFDNSMRLLMLLEFLTNSLQIASLLVQFLMNDDTLVILHVITYIGIVTSQLFLLAWNANEIKEQSSALSVALYESRWYEHGISVRKCILLMMMRSQKPLTMQIGPFYPMTADTAISTMKAAYSYVTIMR

>PverOR38

MMVFFLARIQNPPTITLYKWVNVDMVFYLNVVRMASSFYMLISNVNTK

>PverOR39

MAVLTIMANQKPAKISAGSFVDINLETSLATIKTMVSYCMFLRTMSIDE

>PverOrco

MSGMTYYISLIFQIYYVLFSLTHANLLDSLFCSWLIFACEQLQHLKEIMKPLMELSATLDTYVPKSADLFRAPSANFQDNLIENDYNTKNEELNLKGVYSTRQELGANFRSGALQTFGQGGGGVGPNGLSKKQELMVRSAIKYWVERHKHVVRLVTAIGDAYGVALLLHMLTSTVLLTLLAYQATQINGVNTYAASVIGYLVYSLAQVFHFCIFGNRLIEESSSVMEAAYSCHWYDGSEEAKTFVQIVCQQCQKAMSISGAKFFTISLDLFASVLGAVVTYFMVLVQLK

>PverOR40

MVIKAIPLSAWYPYDEQKHYLPSFCWNVLDGSIGASFLAYSDILSFSLIIFPLGQIHILKHIVANFDDYVKKIENQIGVSQKEASFITIRECILNHNEIIRYIRDLNDAMRNVMLLDFLQSSIQLASLVLQLFEDLILPNIVLFGQFLCSMLIRLLLYYWYSNEITYQSSDIAVAIWNSKWYEQSPKVKNIMLIMMMRCSQKLYLEIGLFSTMSLGTFIY

>PverOR41

MSTLESPHFHYGSCAERNFFPGTTSQRFKKVGITYTMLFYILAHATLISSYLPPLIAAIQHDKNDPEKPLPKRLPYYSWMPFEFNTATLYLIALGYQAIPMFSYAYSIVGMDTLFMNIMNCIVMNLEIVQGAFRTVRERACENTIGPLLTPDELYNTEELGIAMKREMKKINKHLRIIYEMCENLENVYTFLTLSQILATLIILCSCLYLVSTTPTSSKQFAEIIYMIAMGFQLSLYCWFGNEVTLKADRIPFYIWECDWISADTEFKKSMIFTMVRAKRPLHLTAGKFVPLTLSTFIGIIRASYSAYAVIKNTSR

>PverOR42

MFFTRTIIRGMLLRQQDVETSEDLLVMFGSLGATVVCVFFTRSRERWHDFLQCLIDFKPYTSSFEFEEKISERNILGLFYGAYYLMGLIIYGIAAYTDNSTCHRIKEEKNINLFCDTYFPIWIPFDITSRAIHWVIFFVQFFLSTCSFFPAAMICFLAWEATKVICIHINLLKEHFKYIIDNNCRTRPDGLAFWVEYHNHVLQLAETLRVLVKETVGSVALCSAVVLGCNEHSIMRVGI

>PverOR43

MMLLYGDYATLALTFAGIHIFLCVAAFMIFNVHPQSNWRISMSLVPAVIFALNICSNGQRTKDESERVYYNALECGWYNWNIKNRR

>PverOR44

MTNSSILFVEVASLYIQANDVSLESQNLGTNIYESNWYERSPEIKKSLHIMMLRTQQPLKLESGGIVVLGNTLFLKIIKAAYTYILFSNKLN

>PverOR45

MYPMRDQRIFQLLLSWVFLLSPSMSARFSLIFDHSYTICSCRWQY

**GR**

>PverGR3

MIFRLVLISIYAASVNEESKRFLLVLNNVPSELWHHEVERMIGQIDFGGAQLSGYGLFTIDRGLILKVCMTVLSYELILIQFGNSS

>PverGR7

MGRKILDLVQPLFHSVSLLLFTEACDAVEKSGRRIVRTCYSLHKNIQGDALLKEKVKVLARYAEERRPVFCFWNFFHLNRSSLLTFFSAIITYTVIVIQFN

>PverGR9

MLIMLSCLVHLVVTPYFLVAELIKDKSNYFFIFLQVLWIITHTCRLLIIVEPCQACSLEGQRTTMLLCDLLGLSSTDESFRKAVQSFSIYLSQNQIKFSCSGLFTIDRSVI

>PverGR11

MFTRILAMVSVSLLLTEITEGAPAVASSRQFLSGHPGFIPVYIRAGDTPLEDINPELAEAFNSYAQRHGRLTYRRSLEDKSGETGGDEEFPDFAEVGETSLEEDVKPEGDNNKISGSQEASGSHIQKIPRN

>PverGR12

MECLNQTQKTIPIKASRRYALSSIYFLAIVISAFMVLFIFDIIMWYHRGNNNSTYLKEYSTFYFLYAMVVMKEVFFWHVIFLIKIKISLLNNKLLDIKNKAISTDIHFIGLGGNLNTIDNGYKCGNSKDLVKEFIALSSSHKKIVEAVGIVNNSIEFGIHILMLSCLLHLIVTPYFLLMEILSAINDFSNITVQIFWALAHIGRVLIIVEPCQLCINEYEKTLNILCDLATFDFDEEVKKMITLFSLQHSYYKLSFSSCGYFQINRSLLTSIAGAVTTYLVILFQLNNN

>PverGR15

MLAYSIVVFLVVLTYIAYIRWDKVEMFRTAEGRFEEAVIDYLFTVYLVPIVINPIAWYEGRKQARVLTTMMSFEKIYRRVTRKNLSVSLGNTPLVITIGLPALAISAMVVTHVTMVHFKLFQVIPYCYINTTTYLIGGSWYIYCDLIGHVAQTVAADFQQALKNIGPSSRIADYRALWMMLAKLVRDVGNAFAYAVTFLCLYLFLIITLTIYGLLSRIQDGVGIKDVGLTLTAFLSGVLLFFVCDEAHYASNCVKVQFQKKLLLVELNWMNDDAQQEINMFLRATEMNPTDISLGGFFDVNRNLFKSLIATMVTYLVVLLQFQISIPGDDYSEGDASKTNATKIK

>PverGR18

MAHLYGEELHIKQITKFSRQSPRAQEIAKRATLNSADGRVIDKHDQFYRDHKLLLALFRILGVMPIQRGKIGKITFGWLSGPMLYAYVFYAAMTYQVTMVGYERMGILLNESRKFDEYIYSIIFIIFLVP

>PverGR19

MDIVKPRSRKHGPPLSGVLRQQKELLLHHGVLDRVVQVVASLDDLEAALLRRIARHYHDGQRHDHDEGFQEVEDLPAGVVVDGVGEVEEQVQHRDADAVGGDASVEFVEFFDDAVGCFAGVHHFLDWLHACLVFVSGAAQKVVQEVV

>PverGR20

MLLQTLSDELHFQFATTPMVIILDDKFQVSLFLLTEAFDGVEKSGMKIIKTCYTIHKNIQDDVQLKRRVYVLAKYAQKWRPVFSLWNFSRLNRSTSTKFLTAIVTYTVIVIQFNIMMGERTKKQSK

>PverGR21

MLQFSAAMLVLRERFRWINEELKVLIREWKKSCEMKKKDFIVGYVDLRWKSNEEAFIEVLEKLRAKYYDIHDLTIELNAIFSIPAVLTIMQCFLSSLVNILYLVRSVKRRSWYKTVLFRWFGGSILSISAIKLVTISYTSSFLFSEAKKTEAILHQIDSTSEDVNDFVQHFSYQILELKPRITACNLFTIDKSLIYEVVALLTSYLVVCVQLDLK

>PverGR22

MLSIIRSFYTMIQVLVIIFCIDLATREPYKLLKFCINLQGKMPVGSREYQRMELFKRRITELLPKFTAANFFQITRSTILSVISVITTYFIVVVQFYLTMKT

>PverGR23

MAFYLCNCLASIQLIIIARNWSKILGQWSYTEISLRGYPQEENVRRKFLLITLVFMGLGLAEHLLFILNGIFLSKQCVGYEKSHARVYFEVSFQNYFTFIQYNVWLASVIKFANTISTFTWIYTDLFITLISVALTAKFKLITRKLKANTMVHEKFWREMRQDYQKLNNLCMYIEKYLAPLVTVSFVHNIFFLCIQLYNSLSERSGVLETTYFFFSFGFLVLRLMTVSMYGAWLHEETRKPLGFLYRVNTEHYCTDISRWIQQIYVSPTGISGSGFFLVTKPFLLKMAGTIVTFELMLFQFA

>PverGR24

MSCDAVEQSGKKVIQACYDLHHKTTGDERLKSRILMLAIYAEEWRPIFSAAGFYDVNQKCLNSIFSA

**IR**

>PverIR2

MPVNSIIYKVGNTKSSRIVVEALYKFDRTSTNFLTNRIAEWSPENGFRYFRKLISTTNRTNFMGTPIRTSYVIFDNDSLNHLSDYRFIDTDKLSKINYRLIDSLKDLLNTSRVNIWTSSWGNKNLTTGEYSDGIFKDLLSNKADITGTTAFISKDRLDSFTYLVPTTYEEIKFVFRAPSLSYTKNVYTTSFSPMFYLSCLSILVLGGFLLYFLELVEYQCGAIPNRMTLMDVVATQICSITQNSVVRTPELPATRIAVFFNMIFCMLVYTAFSAYIVILLQKTTDEIKDVRSLYDSKMPIGVQNTPYNKYYFSTPNLLTNEHYRKLFYENRLGGSQSNFISPEKGMKNVQGGFYAFQVEQATAHYFIKTTFNENEKCSVKSIPTIFVGFQPYLVIPKNSSYYNHFQVGFRRLFETGLHQREYVRFFTKEHKCQGAQRNYDSVRLMDCYFPFLIFAFGFTLSIFVLMTEKIIKRIGKKGLMQEVCRTKDGYRKRITQPLDFIN

>PverIR5

MKMGLNKIWLVFLSFLLENCQGETTQNINVLFVNEENNGVAEKALDVAMTYLKKNNKLGIAVDLKKVVGNRTDSNKFLEALCSTYNSMLSTQTFPHLVLDMTMTGLGSETVKSFTQALALPTISASFGQEGDLRQWRNINESETDFLIQISPPADVIPEIIRTIVLNQNITNAAILYDSSFVMDHKYKALLQNVATRHIITPIKEVSQLAEQLTQLRKLDLVNYFVLGNLKSIKNVLDAADGLNYFNRKFAWHAITQDDGDIRCTCRNATILFAKPLPNALYQDRLGAMRRTYQLNAEPIVASAFYFDLILHALMAVNEMISDGSWKSGGGGFITCDEYDGSNTPKRGGLELRRFFSKQSSEDPTYGPFSVASNGWSHMEFQMQLTAVGVRDGASDKSVNIGAWWAGFDNNLTLLDAQAMGNLTADVVYRVVTVEQKPFVFRDESSRSGFNGYCVDLIDKIADILKFDYEIVAVDNFGVMDENGKWNGVVKELVEKRADIGLGSMSVMAERENVIDFTVPYYDLVGITILMKLPESPTSLFKFLTVLENEVWLCILAAYFFTSFLMWIFDRWSPYSYQNNREKYKDDEEKREFNLKECLWFCMTSLTPQGGGEAPKNLSGRLVAATWWLFGFIIIASYTANLAAFLTVSRLDTPIESLDDLSKQYKIQYAPLNSSSTQTYFERMANIESRFYEIWKDMSLNDSLSEVERAKLAVWDYPVSDKYTKMWQAMKEAGLPANMAEAVERVRASKSSSEGFAFLGDATDIRYLELTNCDLTRVGEEFSRKPYAIAVQQGSPLKDQFNTAILQLLNRRELERLKEKWWNKNPEKQNCDKIEDQSDGISIQNIGGVFIVIFVGIGLACVTLAFEYWWYKYRKNSKIIDIREAPNAHQDATSKTNPKMRRLFDSKSSDNPTFKNTKAVYPRSRF

>PverIR9

MKRIIQSVVLILFCFGFQMVSSSEVTIGVLLNDLTSQVQLPLNSVIYNKNVFDQNAHFSTDVSLVSNIDSFDASRTLCNMMNSSIGITAVIVEDIPNTIPVLESICTNFEIPFIMTSWRPPVVRNPDQERALLSFYPEAERFAEGLAEIVKSLQWTSFVIVYENEDGLIKMQDVLKLQEYKKNTRRNNIFVKQLGPGPDYRPLLKEIRNTTEDNMILDCKTENILPILLQAKSVNMLNLHNRILITSLNAHTVDFSVLNTTANITILRLHDPKTENFENAIHRWQLTEFENRDIHTQLDPKSIMTETLLFHDAILLLTDAVRDLSITPKIETSPISCSENQTTRDGFAIRNYMRIKTPSMTLTGPLEFNNNGDRIKFNIYAIDIIEDTVIATYFAGNKSITLARSGKESMDAAVLNLQKITVIVSSRLGPPYLMPREPTYEGEEFVGNRRYVGYSMDLIDGIAKIIGFKYEFVITSKYGKYDEEAKRWNGLIGELLEKRAHLAVCDLTITPERTEVVDFSMPFMTLGISILYKKPDKKDINMFGFLETFSKAVWIYTATLYLIISIVLFFISRMTPGDWENPHPCEDEPEELENIWDIKNCLWLTLGSIMTQGCDILPKGISSRMAVAMWWFFSLIMTSSYTANLAAFLTKANLEPEIDGAEALSKQTKIKYGFLAGGSTESFFRNSNFSVYQRMWLNMQQFKPSVFEENNADGVNRVQTTKNSLYAFLMESTQIEYEVETKCTLKQIGNWLDTKSYGIAMPMNSPYRTAINRAVLKMQEAGELGLLKKKWWKDERKEPSCDQNASDDGDSAKLALANVGGVFLVLGVGIAMACIFAILEFLWNVRNITVEEHVSYWEALKVELIFACKVWITKKRTKRLMTESSSSSEKSDRTDDRSIIHSILHSAGSFMHLNAQS

>PverIR10

MAGRETLFFDIQRFGASNFHLSEKLNTAYSAIALQLGCPYTEEINKILTAIFEAGIITKMTENEYEKLGKQKELTSDIAES

>PverIR11

MHLPHADSARILVGSWWLVVLVIATTYCGNLVAFLTFPKIDIAISTVEDLVTHKDTVSWSFREGSFLEDKLQTA

>PverIR12

MKKRFLETGKCEFTLSTEDFFEEELALILPQESPYLARINEEIKRLHQVGLIHKWLQDYLPKKDQCWKNRNIVEVKNHTVNMDDMQGIFFVLFLGFMLSLVLLLFETIWKKHFRKKQQIIIHPFTT

>PverIR13

MVLPVRRSLTIIQSVSIISRIGDGVHFHGIVDVPHEVTFERVFSPIEDINGVSAYEISVVNLLQ

>PverIR14

MTTISAEEVHHLPTMRLGAALLVTTLATLLVESHLLESNQEAKWADSFLKGTKIESTLHIVELLKYIALNYLTDCTPMILFDSSEKNDDLFIEKLLTQFPIPYFHGRISDSYEMMMGSDSAPTTCVSYILFLTDVMRCRNIIGDISHQRVVVIVRSSQWRVLDFLMHEDSRVFMNILVIVKSERIVSPQKPIFQEAPYILYTHELYADALGSSRPIVLTSYQHGRFTRLVNLFPKKMSTGFSGHRFVVAVAHQPPFVSREKFSDGDKKFVGIEVGLVEMLSRIYNFTTDYREATDDIVLGSVQISRSGFQFILCTEKTPSSL

**SNMP**

>PverSNMP1a

MLQMINLGPGTDIRDMFLKVPFPLTYRVYIFNVTNPDRIQKGDMPVVNEVGPFCYEEWKEKMNVEDMEADDTIAYDPKDTFLKKRWPGCKTGKEIITVPHPMILGLVNTVARQKPGALSLANKAIKSIYANPSSIFITTEADNILFDGVIINCGVTDFAGKAICSQLKSSGNLKLINGDQLLFSLLGPKNATLNTRMKAYRGKKHFQDVGRIVEFGGAKNLDVWPTDECNEIKGTDGTIFPPFLKKEQGLVSYSPDLCRSLRATFVKDTVYDGIPCAEFTATLGDMSKNEDEKCYCLTPDTCMKKGIMDLYKCAGVPVYASLPHFYGTDKSYLDGVNGLTPNKSKHEIKILFESTTGSPLYARKRIQLSMPLEPIQKVELFMNFTPTVIPVLWIEEGVELNRTYTGQLKSLFTMKKIVGAFKWVVLLSSLGGLAAAGYMFYKNNGKIEITPIHESKRDGISTIHSLEGQVNHGMSENSIDKF

>PverSNMP2a

MLACSRFFSNKVLAVLTTILTLIFVGVLVLAFYGIPVIINKSIHNSVHLEKGTIQWDRFVDLPVDILMKVFLYHVTNSDDVLNGAKPIVEERGPYCYKQNIHKNILSTSSSQDTVTYEQNFKIEFDQEASGNLKESDKVVIVNPVMLTLYKLTSRLERLVVFGCLDKIFPKEYIGVFIEVDVKTVMFDGFAFAQRSEDLGPACNIVRNQILDKTLPMKNVERITDDDGILELRFALLQYKIRGPDGNYTINRGIDDITKLGHIIKWNDETELPFWGRMQSINNDTCKKVRGSDSTIYPPQVDKTRSFDIFSTDICRVVEISFQRTDTYNGIDAYRFGITKNTFRSATTNPENDCYCIKQSAGIDGEPSCYLDGVLDVYPCFGAPILLSFPHFLYADESYVDAIEGIGPSDPDIHELFLLIEPNTGTPLQGMKRVQLNTVLMPMQNIPGTSKISPLVMPILWLEEGVSLPQNLIDELNSHYFQTVKLVEGIIYGLIAVVAASVLISSGFLIRRKCC

>PverSNMP2b

MVFNLKFNLSKKTLKILGCFGLFLACAGVYFGYKALPDIVTDKIWEMKVLKENTQQWDMFKKMPFPFTFKVFIFDIKNPDEIMQGAKPTIKEIGPFVYKVYKWNSDIKWESPDDISYFAYTRFEFDEEASGRFTEDYIVTILNTPYLGMLLKVADIQAAALPMVEGVLGDIFKENDGLFIKVKVKDYLFQGLKMCENEGKDGDFAAGLVCKQVIAEAATSNNLRVENNTILFANLHYKNNTHLGRFTIKAGIQNHNEIAHLALYNNQSYISIWGEEKSICNKIEGLSTTVFPVNINKDMIFESFAEDICRRMKLTYKMDETVKGLKGYKFTAANDSFSMKNENNTCYCNKKTTLMDGKLGCVKDGITDLSTCTGGPVMVSFPHLLYADKEYLNSVEGLDPDSMKHESFVVLEPMSGFPLSLAQRVQFNIFLRPIDESTILANVSRALFPLIWVEESLQLDDKFTDMLKNNLFKTLDMINILKWVVIASGSACFLFAVSMAIYNDAS

>PverSNMP1b

MQLAIKVLVSGVVITLSSVIFALVIYDPLIKYVIRDQTSLKKNNQIRDIYLKIPFPLDFRIYLFNVSNPMEVQDGAKPVLKEVGPYCYDEYEEKVDVIDNEMEDSLTYNSYDIFRFNANKSIGLSENDYVTIIHPLIVAMAYQVNRDTPALLSFLNQAIVTIFKNPKSIYLTDTVKNILFDGFEINCNVTEFAAKAVCTQIMNSNIPGLKTDPSRNNTLVFSLFGARNATLGHTMKVLRGIKRSEFVGKVLEVDGKKEMNLWTSKACNRYRGTDGWIIPPLLEPGVGVWTHSVDMCRNVEAKYIKETVLNGVNARLYEADLGDMQKNEDEKCYCPTPSTCSRKGTFDLTKCMGAPIIASLPHFLRADEIYRQQVDGMQPVHEKHIISIYLEGVTSAPLRATKRMQLNFPITTIPKLTLMTKLPEALHPLLWLEEGVEVEGEFLKLITDKLMLLNVANYGRWLAVFGGLITTGVGVYLHNKNKNSVAISTIHSGDIDREITRSTNELMDQMNRIQGNEKGHVN
